# Transcriptomic analysis of circulating extracellular vesicles during the perioperative period of Fontan and Glenn surgery

**DOI:** 10.1038/s44325-024-00039-1

**Published:** 2024-12-18

**Authors:** Felipe Takaesu, Khalid Yasseen, Evan Yang, Hyun-Ji Park, John M. Kelly, Christopher K. Breuer, Michael E. Davis

**Affiliations:** 1 Wallace H. Coulter Department of Biomedical Engineering, Georgia Institute of Technology & Emory University, Atlanta, GA USA; 2 Biochemistry, Cell and Developmental Biology Graduate Training Program, Graduate Division of Biological and Biomedical Sciences, Laney Graduate School, Emory University, Atlanta, GA USA; 3 Frank H. Netter School of Medicine, Quinnipiac University, North Haven, CT USA; 4 Department of Molecular Science and Technology, Ajou University, Suwon, 16499 Korea; 5 Center for Regenerative Medicine, Abigail Wexner Research Institute at Nationwide Children’s Hospital, Columbus, OH USA; 6 The Heart Center, Nationwide Children’s Hospital, Columbus, OH USA; 7 Department of Pediatrics, The Ohio State University College of Medicine, Columbus, OH USA; 8 Children’s Heart Research & Outcomes (HeRO) Center, Children’s Healthcare of Atlanta & Emory University, Atlanta, GA USA

**Keywords:** Congenital heart defects, Cardiovascular models

## Abstract

Single-ventricle defects are treated with the Glenn and Fontan procedures, which offer lifesaving relief but result in lifelong complications. To address the lack of outcome predictors, we conducted an untargeted transcriptomic analysis to identify RNA biomarkers in serum and circulating sEVs from 25 Glenn or Fontan patients with three samples exclusively used for experimental assays. Unsupervised analysis revealed a distinction between pre-op and post-op samples in both surgical groups. Differential gene expression and pathway analysis showed enrichment for pro-angiogenic cargo in post-op sEVs compared to pre-op sEVs. Wound healing assays revealed post-op Fontan sEVs induce a stronger pro-angiogenic response than pre-op Fontan sEVs. A PLSR-guided approach revealed MAPK6, GLE1, hsa-miR-340-5p, and hsa-miR-199b-5p as key transcripts in the observed wound healing response. Lastly, EV-Origin revealed decreased secretion of sEV from cardiac tissue and increased secretion from brain tissue for both Fontan and Glenn samples. This work demonstrates the potential of sEV RNAs as biomarkers for patients with Fontan physiology, enabling quicker diagnosis for Fontan-associated complications.

## Introduction

Single-ventricle defects comprise a spectrum of malformations that generally result in one, instead of two functioning ventricles^1^. These defects can be palliatively managed through a series of surgical procedures, in what is commonly referred to as Fontan palliation. The initial palliative intervention varies based on the defect type, but the procedures culminate into the Glenn (at ~6 months of age) and Fontan surgeries (2–4 years of age)^2^. The goal is to use the single functioning ventricle to systemically pump blood while rerouting the systemic venous circulation directly to the pulmonary arteries without the use of a sub pulmonary pump. The resulting circulatory physiology is characterized by significantly elevated systemic pressure and low normal cardiac output and will be referred to herein as Fontan physiology^3^. Although lifesaving, we now appreciate that patients undergoing these procedures suffer from significant end organ dysfunction which leads to increased morbidity and mortality^2,4^. While it is believed that this dysfunction results from abnormal hemodynamics, there are currently no known mediators for these adverse outcomes, and surveillance is primarily focused on identifying end-organ damage^4^. Thus, the need to identify novel biomarkers to predict complications early in the Fontan physiology is crucial to help determine the timing of intervention and to better understand the diverse complications which arise over time.

Analyzing the circulating transcriptome is a promising method to identify novel biomarkers in the Fontan physiology. Current efforts to identify non-invasive diagnostic markers use a targeted proteomic approach to correlate serum protein levels to post-Fontan clinical symptoms^5^. Although protein-focused methods have experienced some success, few studies investigate the potential of other molecular species like RNAs as possible biomarkers. These circulating RNAs have been shown to act as strong predictors for clinical outcomes and exist either as free molecules or enclosed within vesicle-like structures such as small extracellular vesicles (sEVs). sEVs are nano-sized lipid membranes released into the interstitial space that contain cargo consisting of DNA, protein, lipid, and RNA^6^. Evidence shows that sEVs can act both as highly sensitive biomarkers and also as potent mediators for adverse outcomes through direct cargo delivery to recipient cells^7^. As a result, sEVs are thought to reflect the physiological status of the body and are a suitable vehicle for identification of novel biomarkers in single-ventricle patients. Thus, elucidating the circulating sEV transcriptome in the Fontan physiology can not only identify novel, highly sensitive biomarkers, but it can also provide further insight into systemic changes that occur through staged palliation^8^.

Given the potential of sEVs in diagnosing post-Fontan complications, we hypothesized that the Fontan operative sequence triggers a change in the circulating sEV transcriptome in patients and such changes can serve as valuable predictors of post-Fontan complications. To investigate our hypothesis, we collected and analyzed RNA from serum and serum-derived sEVs from a cohort of pediatric patients during the perioperative period of the Fontan and Glenn procedure. First, we compared the preoperative and postoperative transcriptome of serum and sEVs from both Glenn and Fontan patients and found distinct transcript signals originating from each. An in vitro wound healing assay revealed that Fontan post-op sEVs have a higher angiogenic potential than pre-op sEVs. Thereafter, we utilized a partial least squares regression model to identify key RNA drivers of angiogenesis, a key molecular driver of venovenous collateral formations. Lastly, we leveraged a tissue deconvolution tool to identify changes in sEV expression in preoperative and postoperative samples in comparison to a previously published sheep Fontan model. This study highlights the potential of serum and sEV RNA-based biomarkers and supports them as mediators for the formation of venovenous collaterals in patients with single-ventricle physiology, paving the way for the discovery of long-term RNA-based biomarkers.

## Methods

### Ethics

Blood samples were collected under an approved IRB (00005500) prior to surgery and before discharge from patients undergoing Glenn or Fontan surgery. Informed consent was received from parents prior to study enrollment.

### Statistics

*P-*values for differentially expressed genes were corrected for multiple testing using the Benjamini-Hochberg adjustment. Genes with a |log_2_FC| greater than or equal to 1 and an adjusted *p*-value less than or equal to 0.05 were considered differentially expressed.

For the wound healing assay, a two-way repeat measures ANOVA with Tukey posthoc was performed to assess differences between pre-op and post-op (Glenn or Fontan) samples to the negative control. An adjusted *p*-value less than or equal to 0.05 was considered statistically significant. RT-qPCR mean expression differences were compared using a one-way ANOVA with Tukey posthoc. An adjusted *p*-value less than or equal to 0.05 was considered statistically significant. GraphPad PRISM 8 software (GraphPad, San Diego, CA) was used for statistical analysis for both wound healing and RT-qPCR experiments. For miRNA inhibition experiments, a paired Student’s t-test was used to assess differences in wound closure between groups with a *p*-value less than 0.05 being considered significant.

### Serum sample collection

Human blood samples were collected under an approved IRB before discharge from patients undergoing Glenn or Fontan surgery. Sex and race were determined based on clinical registry. Samples were clotted using a serum blood collection tube (BD Vacutainer, Cat # 02-683-94) for 15 min at room temperature. Samples were then spun at 1500 × *g* for 15 min at 4 °C followed by immediate serum collection from the supernatant. Serum samples were stored at –80 °C for long-term use.

### Sheep animal model

Details on the development of the sheep Fontan model are available in our group’s previous work^8,9^. In brief, the sheep SVC was directly anastomosed to the main pulmonary artery and the IVC was connected to the pulmonary artery with an ePTFE graft. Venous blood samples were drawn from the internal jugular vein of the sheep preoperatively and up to one week postoperatively. Sheep subjects ranged between one to two years of age, an equivalent of early adolescence and early adulthood in humans. Sheep serum samples were stored at –80 °C for downstream applications.

### Extracellular vesicle isolation and characterization

Serum samples were thawed on ice in preparation for sEV isolation. 1000 µL of serum was centrifuged at 10,000 × *g* for 10 min to remove large particles and debris. 500 µL of supernatant was loaded into a qEV 35 nm original column (Izon Science, Christchurch, New Zealand, Cat # ICO-35) to isolate sEVs. sEV fractions were eluted in 1 mL of PBS and stored at –80 °C for downstream applications. sEV size and concentration were measured via nanoparticle tracking analysis using a NanoSight NS300 (Salisbury, UK). In brief, sEV samples were diluted 1:50 in PBS, and 1 mL of sample was injected into the NanoSight at a flow rate of 500 µl/min. Three 60 s videos at different sample locations were gathered for size and concentration analysis. Additionally, sEV morphology was also characterized by negative stain and transmission electron microscopy imaging through the Emory Integrated Electron Microscopy Core.

### RNA isolation and sequencing

In total, 300 µL of serum or 700 µL of EVs were used to isolate RNAs using a Plasma/Serum RNA Purification Mini Kit (Norgen Biotek, Canada) and an Exosomal RNA Isolation Kit (Norgen Biotek, Canada, Cat # 55000) per the manufacturer’s instructions. Quality control of the isolated RNAs was performed using an Agilent Bioanalyzer (TapeStation 4200, Agilent Technologies). miRNA and total RNA library preparation and next-generation sequencing were done by the Yerkes Nonhuman Primate Genomics Core at Emory University.

### RNA alignment

Raw data from Illumina was converted to fastq format. Total RNA sequences were cleaned using the TrimGalore wrapper package. Illumina adapters from 3’ and 5’ ends were trimmed using the TrimGalore subpackage, cutadapt, and reads with less than 15 base pairs were removed. Additionally, reads with average quality score below 20 were removed. Cleaned reads were then further processed using the bbduk.sh package in the BBMap suite. Reads were analyzed for polybase sequences and ribosomal RNA contaminants and removed. The subsequent reads underwent alignment using the STAR aligner to GRCh38 for human total RNA and OviAri4 for sheep total RNA^10^. miRNA sequences were processed using a similar pipeline. QIAseq miRNA adapters from 3’ and 5’ ends were trimmed using the TrimGalore wrapper package, followed by quality trimming. Alignment of the miRNA was performed using miRDeep2^11^.

### Differential gene expression analysis

Aligned reads were filtered using the filterByExpr function from the edgeR package in R to remove lowly expressed genes and normalized with the trimmed mean of M-values (TMM) method^12^. Principal component analysis was performed using the prcomp function from the stats package in R. Heatmaps were made using the pheatmap function with a euclidean distance calculation and a ward.D2 clustering method. Differential gene expression of serum and serum-EVs was done using the dream (differential expression for repeated measures) linear mixed modeling approach to account for paired sequencing data^13^. Weights were estimated using the voomwithDreamWeights function using default parameters. Sample grouping between pre-op vs post-op was treated as a fixed effect while sex and race were treated as random effects.

Data mined GEO data were filtered using the filterByExpr function and normalized with the TMM method from the edgeR package. Differential gene expressions were done using the limma-voom workflow with sample group (pre-op vs post-op) treated as a fixed effect. |log_2_FC| greater than or equal to 1 and an adjusted *p*-value less than or equal to 0.05 were considered differentially expressed.

### Pathway and protein-protein interaction analysis

For total RNA pathway analysis, differentially expressed genes were input on Metascape as a multiple gene list to compare pre-op to post-op^14^. Only GO Biological Processes were considered for downstream analysis. Target genes from VIP miRNAs were identified using miRTarBase^15^. Targets were defined as those having been validated by at least three different experimental methods. Protein-protein interactions were determined using the STRING (v.12.0) database^16^. Searches were limited to *Homo Sapiens* protein hits and the required score was set to 0.900 (highest confidence). Textmining was excluded as one of the active interaction sources.

### sEV tissue origin

sEV tissue origin was determined by the EV-Origin algorithm published by Li et al.^17^. Briefly, sEV reads were TPM (transcripts per million) normalized. Differences in estimated tissue fractions were used to compare pre-op and post-op human and sheep samples. To compare our results with previously published data, we mined circulating sEV RNA data with a similar experimental design to ours from the Gene Expression Omnibus (GEO). When needed, gene names were changed to human gene names using the biomaRt package in R^18^.

### HUVEC culture

HUVECs were cultured in endothelial growth medium (EGM-2, Lonza, Bend, OR, Cat # CC-4176) supplemented with 2% FBS, 1% penicillin-streptomycin, 0.4% hFGF-β, 0.1% vascular endothelial growth factor (VEGF), 0.1% ascorbic acid, 0.1% long arginine 3 insulin-like growth factor (R3-IGF-1), 0.1% heparin, 0.1% human epidermal growth factor (hEGF), 0.04% hydrocortisone, and 0.1% Gentamicin/Amphotericin-B (GA-1000), per the manufacturer’s instructions.

### Wound healing assay

HUVECs were cultured to 80% confluency with endothelial cell growth medium and then seeded on 12-well plates with 1 × 10^5^ cells per well. Cells were incubated for 48 h until they formed a monolayer. A P1000 pipette tip was used to gently form a scratch on the monolayer followed by two PBS washes. Brightfield images were taken as an initial assessment of the wound size using a phase contrast microscope (Olympus IX71, Olympus Center Valley, PA). Cells were then treated with endothelial basal medium (EBM-2, Lonza, Bend, OR, Cat # 00190860) with 20 µg of pre-operative sEVs (*n* = 8 biological replicates with 3 technical replicates) or post-operative sEVs (*n* = 8 biological replicates with 3 technical replicates) and incubated for 16 hours. PBS was used as a negative control. After 16 hours, plates were imaged again to quantify wound closure.

miRNA inhibition experiments were performed using similar methods. Lyophilized miRCURY LNA miRNA inhibitors (Qiagen, Hilden, Germany) for hsa-miR-340-5p and hsa-miR-199b-5p were reconstituted with 1X TE Buffer following manufacturer’s instructions. HUVECs were cultured to 80% confluency and seeded on 12-well plates with 1 × 10^5^ cells per well. Cells were incubated for 48 h until they formed a monolayer. A P1000 pipette tip was used to form a scratch on the monolayer followed by two PBS washes. Brightfield images were taken as an initial assessment of wound size. For transfection, a 1:1 mixture of re-suspended inhibitor and EBM-diluted Lipofectamine 3000 (Thermo Fisher Scientific) was used. Cells were treated with endothelial basal medium containing either 20 µg of sEVs, or 20 µg of sEVs and 10 nM miRNA inhibitor. After 16 hours, plates were imaged again to quantify wound closure. Images for all wound healing experiments were analyzed with ImageJ (Fiji, National Institutes of Health, Bethesda, MD) using the wound healing size tool with manual input^19^.

### sEV RNA uptake and RT-qPCR

HUVECs were seeded in a 6-well plate at 5 × 10^5^ cells per well and incubated overnight to 80% confluency. A P1000 pipette tip was used to form a scratch followed by two PBS washes. HUVECs were incubated with EBM and one of the following: 100 µgs of pre-op, post-op sEVs or a PBS equivalent. After eight hours, cells were washed to remove unbound vesicles and collected via trypsinization. RNA extraction was performed using a PureLink RNA Mini Kit (Invitrogen, Waltham, MA). RNA concentration and purity were assessed using a NanoDrop One (Thermo Fisher Scientific). Reverse transcription of 200 ng total RNA was performed using a High-Capacity cDNA Reverse Transcription Kit (Applied Biosystems, Waltham, MA) following the manufacturer’s protocol. Quantitative PCR was performed using PowerUp SYBR Green Master Mix (Thermo Fisher Scientific) in a StepOnePlus system (Applied Biosystems, Waltham, CA). Primers were designed using PrimerBank software and specific primer sequences can be found in Supplementary Table 7^20^.

### Partial least squares regression

Partial least squares regression (PLSR) analysis was performed using SIMCA-P software (version 16, UMetrics, Sartorius Stedim Biotech, Umeå, Sweden) as previously described to solve the PLSR problem with a nonlinear iterative partial least squares algorithm^21,22^. mRNA and miRNA data were normalized for library size, log-transformed, scaled and centered before being incorporated into the PLSR model.

## Results

### Study design and small extracellular vesicle characterization

**Fig. 1:**
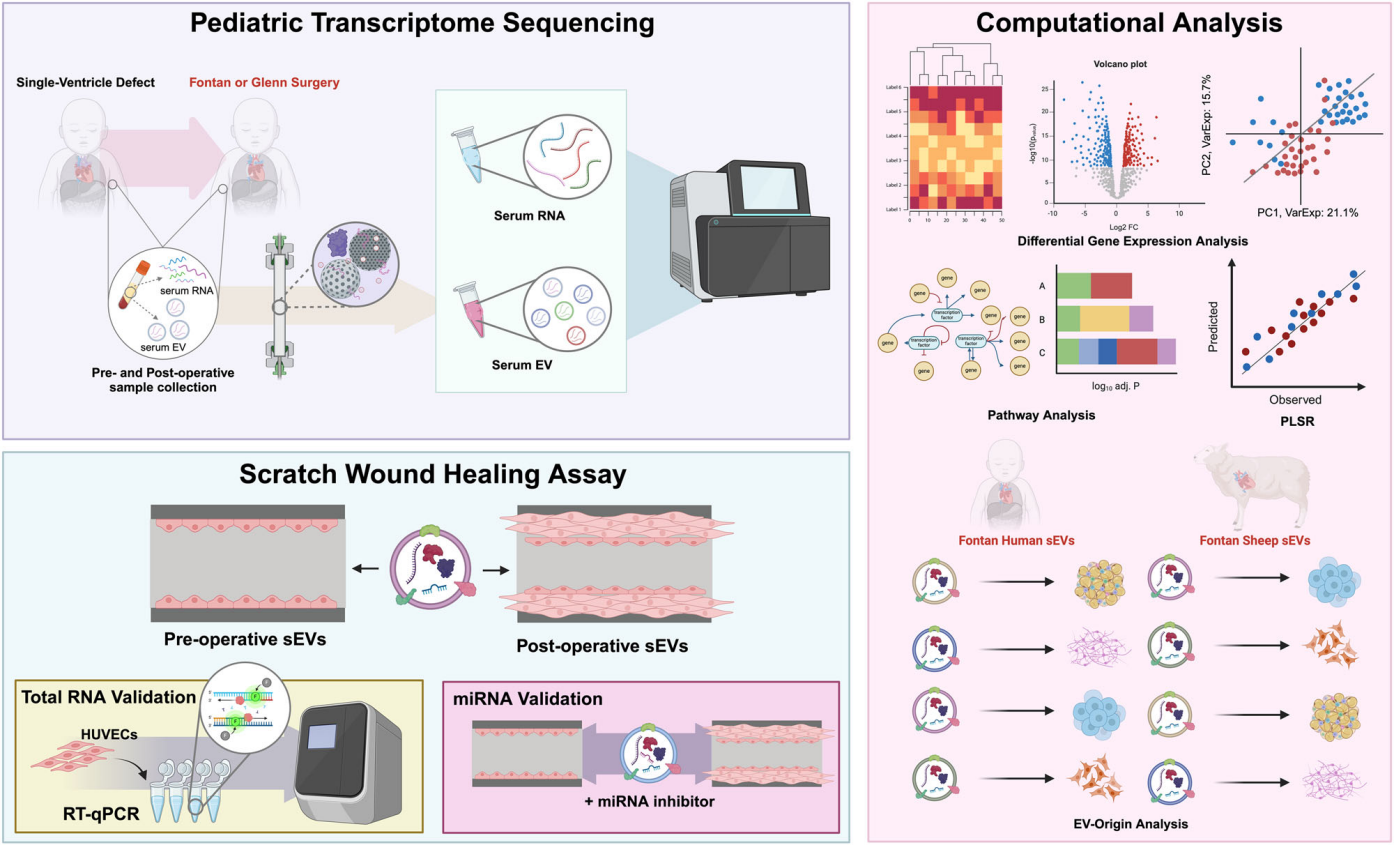
Graphical representation of the study design. Blood samples were collected from pediatric patients with single-ventricle defects undergoing either the Fontan or Glenn procedure (top left panel). Serum was separated from the blood following centrifugation and circulating sEVs were isolated using size-exclusion chromatography. RNA was extracted from both serum and sEVs for sequencing sent. Sequenced reads were aligned to the GRCh38 genome for downstream analysis (right panel). To uncover clustering patterns distinguishing pre- and post-operative sEVs, unsupervised hierarchical clustering and principal component analysis were employed. Differential gene expression analysis was conducted to identify significantly up- and downregulated genes. Results from pathway analysis of these genes motivated subsequent scratch wound healing assays (bottom left panel), which were used to assess the pro-angiogenic potential of pre- and postoperative sEVs. Key RNA cargo contributing to the observed phenotype was identified through RT-qPCR and miRNA inhibition experiments. Finally, EV-Origin deconvolution analysis was performed to delineate tissue-specific sEV responses following Fontan or Glenn surgery (bottom of right panel). A sheep Fontan model and four publicly available datasets were referenced to distinguish Fontan- and Glenn-specific molecular signatures.

Blood serum was collected from patients with single-ventricle defects undergoing either Fontan or Glenn surgery during the perioperative period. A complete list of patient characteristics and clinical diagnosis can be found in Supplementary Table 1 and 2. A total of 25 patients were enrolled in the study with 13 out of the 25 patients undergoing the Fontan procedure and 12 out of the 25 patients undergoing the Glenn. A combined 40 serum samples and 35 sEV samples were used for analysis due to low sEV and serum RNA yields on four and nine samples, respectively (Fig. 1). 3 samples (Patients 24, 25, and 28) were used exclusively for sEV assays. Samples taken before surgery were labeled as preoperative (pre-op) and samples taken after surgery were designated as postoperative (post-op).While serum RNA alignment metrics were lower than anticipated, this is in-line with previous serum clinical reports (Supplementary Table 3, 4)^23^.

**Fig. 2:**
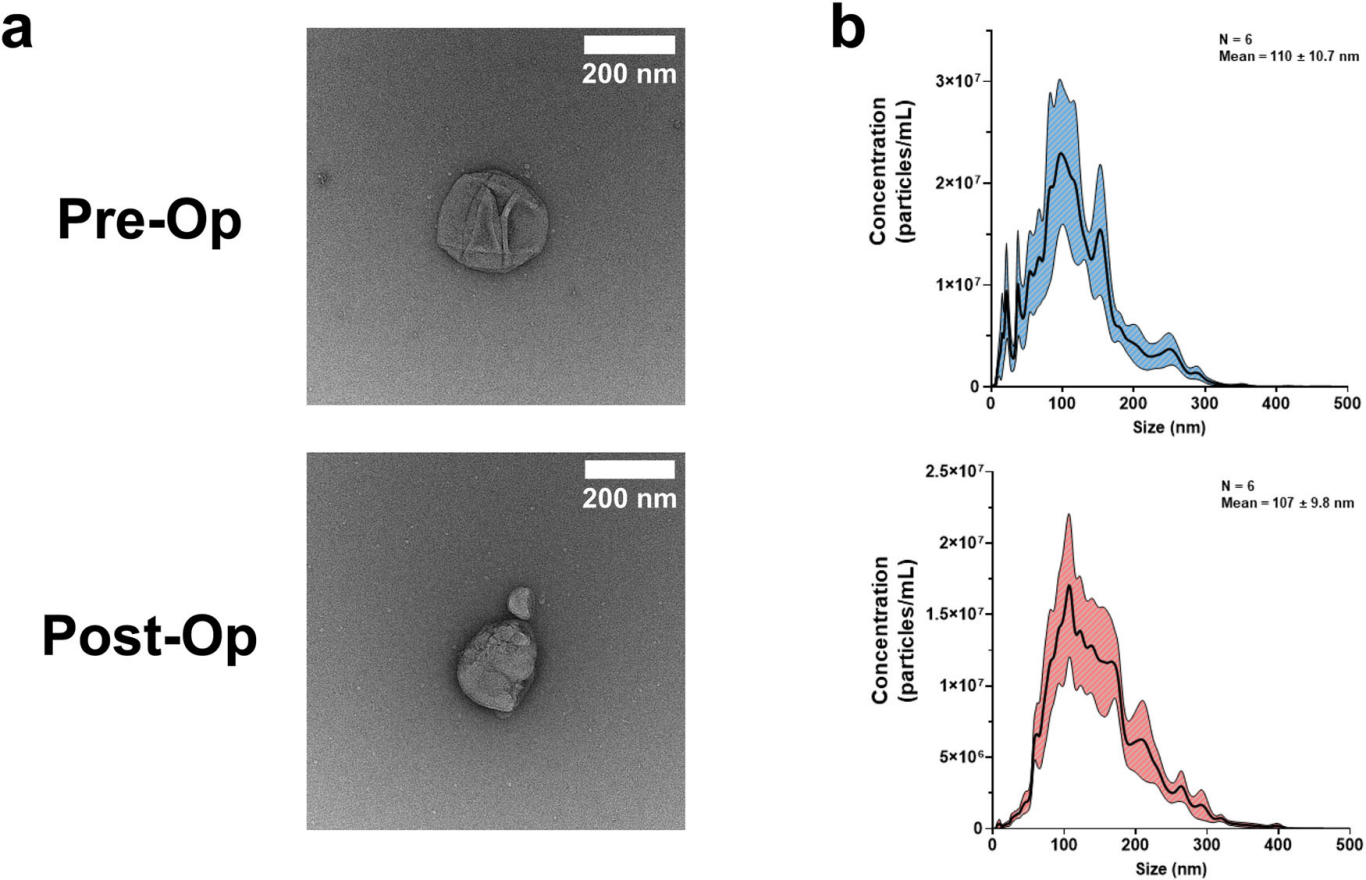
sEV morphological characterization. **a** Representative negative stain transmission electron microscopy (TEM) images of pre-op and post-op sEVs. TEM images show canonical cup morphology in both pre-op and post-op sEVs revealing successful isolation of sEVs. **b** Nanoparticle tracking analysis of pre-op sEVs (mean size = 110 ± 10.7 nm, *n* = 6) and post-op sEV (mean size = 107 ± 9.8 nm, *n* = 6) samples revealing that our isolated sEV samples fall within the acceptable size range for sEVs. Particle concentration was multiplied by 50X to account for the dilution factor.

We characterized the size and shape of isolated sEVs via negative stain transmission electron microscopy (TEM). TEM images showed a canonical sEV double-membrane and globular shape for both sample types (Fig. 2a). Additionally, we measured the sEV size distribution and concentration via nanoparticle tracking analysis. Both sample types had similar size ranges with pre-op sEVs having a mean size of 110 ± 10.7 nm and post-op sEVs having a mean size of 107 ± 9.8 nm (Fig. 2b).

### Unsupervised transcriptomic analysis of serum and circulating extracellular vesicles

**Fig. 3:**
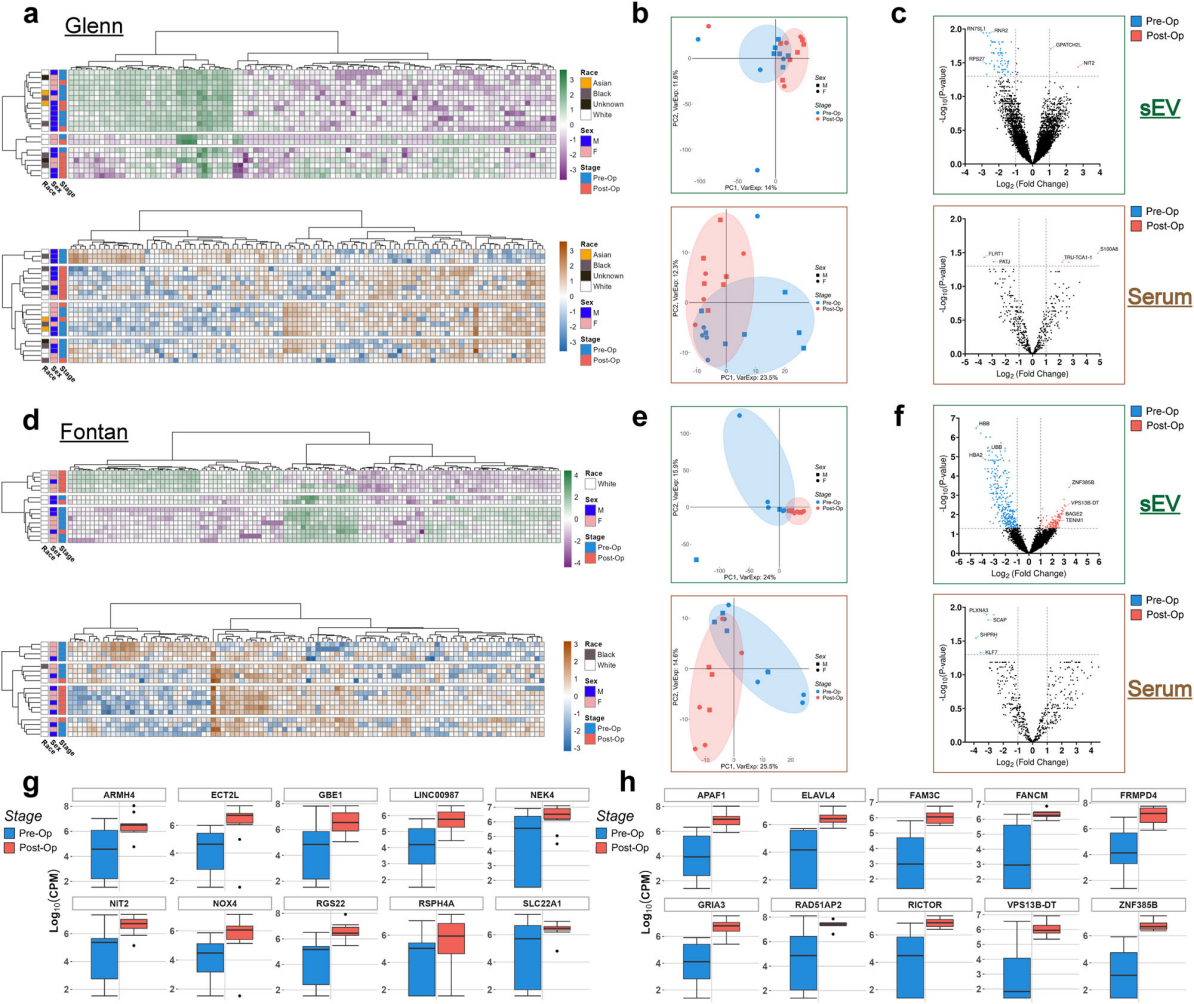
Unsupervised and differential gene expression analysis of Fontan and Glenn serum and sEV samples. **a** Unbiased heatmap of top variable RNAs in Glenn sEV (top) and serum (bottom) samples reveals significant clustering between pre-op and post-op samples. The top 1% variable genes were used for sEVs while the top 100 genes were used for serum samples. **b** Principal component analysis of Glenn sEV (top, green) and serum (bottom, brown) datasets showing tight clustering between pre-op and post-op samples. Surgery type and surgical stage were used as clustering parameters. **c** Volcano plot of sEV (top, green) and serum (bottom, brown) samples highlighting differentially expressed RNAs after the Glenn procedure. Genes with a |log_2_FC | ≥ 1 and an adjusted *p* value ≤ 0.05 were considered differentially expressed. Three differentially expressed genes were observed in post-op samples for both datasets while 76 and two were found in pre-op samples for sEVs and serum, respectively. **d** Unbiased heatmap of top variable RNAs in Fontan sEV (top) and serum (bottom) samples showing significant clustering between pre-op and post-op samples. Race and sex were not significant factors in clustering. **e** Principal component analysis of Fontan sEV (top, green) and serum (bottom, brown) datasets with significant clustering between pre-op and post-op samples. **f** Volcano plot of sEV (top, green) and serum (bottom, brown) samples highlighting differentially expressed RNAs after the Fontan procedure. Genes with a |log_2_FC | ≥ 1 and an adjusted *p* value ≤ 0.05 were considered differentially expressed. 132 differentially expressed genes were observed in post-op sEV samples while none were detected for serum post-op. 330 and six genes were found to be differentially expressed in pre-op samples for sEVs and serum, respectively. **g** Boxplot of the top 10 differentially expressed genes in post-op Glenn sEVs. **h** Boxplot of the top 10 differentially expressed genes in post-op Fontan sEVs.

We first examined transcriptome variations between pre-op and post-op samples from Fontan and Glenn patients through clustering and differential gene expression analysis. Hierarchical clustering of Glenn sEV and serum samples revealed a clustering pattern separating pre-op and post-op samples irrespective of sex or race with larger variability present in sEV samples (Fig. 3a). Principal component analysis of our datasets showed similar separation patterns with the top two principal components accounting for 25.6% and 35.8% of the variance in sEV and serum datasets, respectively (Fig. 3b). sEV variance was primarily captured by the top six principal components while serum variance was primarily captured by the top three principal components. Clustering patterns for both serum and sEV samples were consistent across the top principal components when analyzed together (Supplementary Fig. 1a). Differential gene expression analysis between pre-op and post-op sEV Glenn samples identified 76 and three differentially expressed genes, respectively. Serum samples had fewer differentially expressed genes, with two in pre-op and three in post-op samples (Fig. 3c).

We analyzed our Fontan samples using the same pipeline. An unbiased heatmap of top variable genes demonstrated distinct clustering between pre-op and post-op samples, independent of sex, in both sEVs and serum. (Fig. 3d). A principal component analysis revealed a visible separation between pre-op and post-op samples in serum but with more overlap in sEVs (Fig. 3e). For both, the first two principal components accounted for approximately 40% of the total variance in the dataset. Like Glenn samples, clustering patterns for sEVs and serum were consistent across the top principal components (Supplementary Fig. 1b). However, unlike the Glenn samples, we detected a larger number of differentially expressed genes in Fontan sEVs with 330 and 132 genes found in pre-op and post-op samples, respectively. Fontan serum samples, however, had six differentially expressed pre-op genes and no differentially expressed genes in post-op (Fig. 3f). Plotting the top differentially expressed pre-op and post-op sEV genes for both Fontan and Glenn showed significant variability in the pre-op samples (Fig. 3g, h). Less variance was observed in the top differentially expressed genes for serum samples in both Glenn and Fontan samples (Supplementary Fig. 2a, b). To account for the possibility that changes in the sEV transcriptome could result from surgery rather than the Fontan or Glenn procedures specifically, we mined four published sEV total RNA-sequencing datasets from the Gene Expression Omnibus with similar experimental designs as our study^24–27^. Our comparison found that our top differentially expressed genes are uniquely up-and-down-regulated for both Fontan and Glenn samples (Supplementary Fig. 2c, d).

### Post-Op sEV cargo enhances endothelial cell migration

**Fig. 4:**
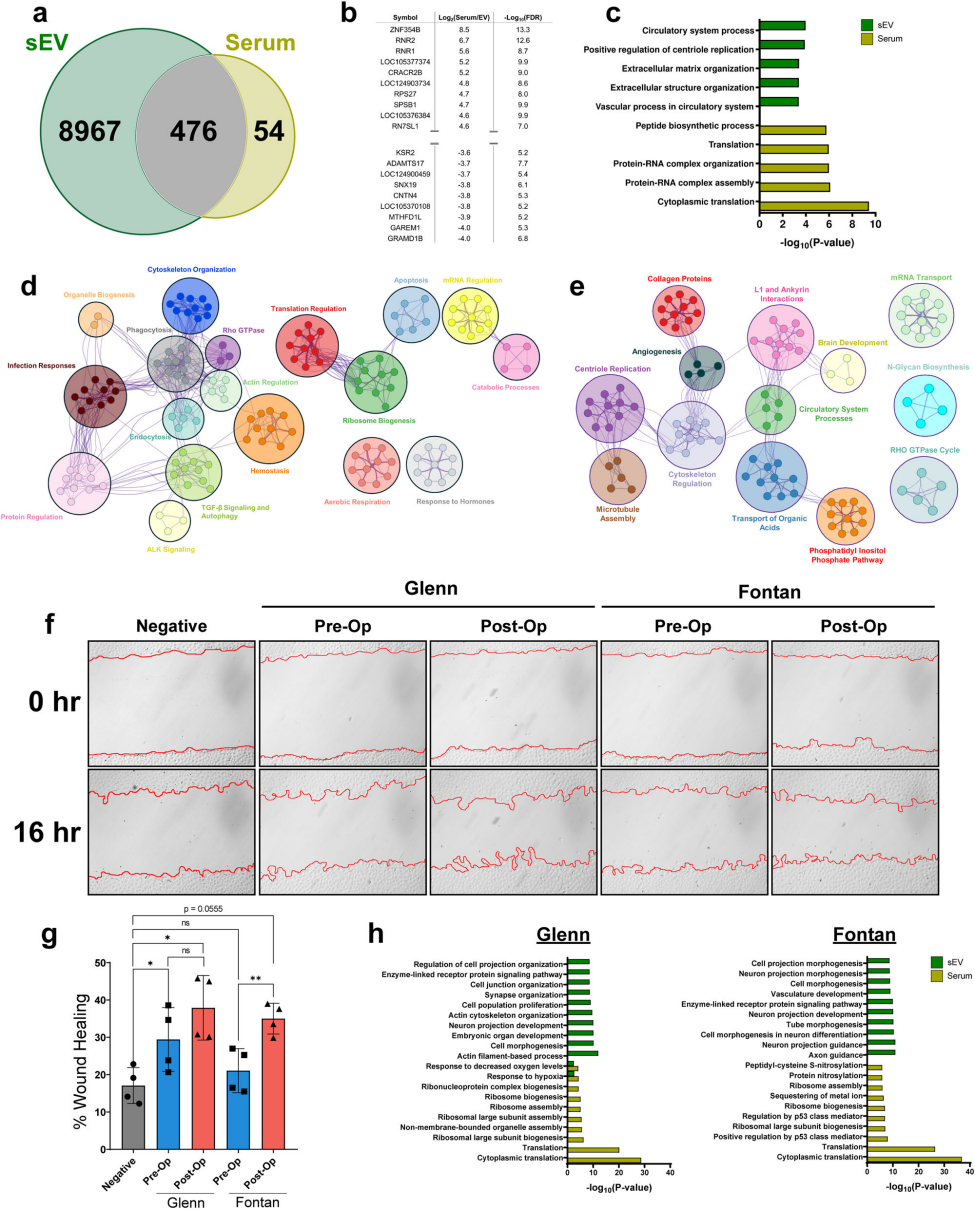
Validation of post-op sEV pro-angiogenic cargo via wound healing assay. **a** Venn diagram of RNAs found to be expressed in sEV and serum. **b** Top table of differential gene expression analysis between serum and their respective sEVs. **c** Metascape pathway analysis of differentially expressed genes found in sEVs and serum. sEVs are enriched for extracellular matrix organization and circulatory processes RNAs while the serum transcriptome is enriched for RNAs regulating translation. **d** Cytoscape pathway interaction network of pre-op sEVs reveals an enrichment for pathways involved in cytoskeleton organization, ALK signaling, aerobic respiration, apoptosis, ribosome biogenesis, and protein regulation. Clusters were assigned categories based on common pathways found in each cluster. **e** Cytoscape interaction network of post-op sEVs shows an enrichment for pathways involved in microtubule assembly, cytoskeleton regulation, collagen proteins, and angiogenesis suggesting a more potent pro-angiogenic cargo than pre-op sEVs. Clusters were assigned categories based on common pathways found in each cluster. **f** Representative wound healing images comparing pre-op and post-op sEVs from Fontan or Glenn samples. Images were taken immediately after the scratch wound and 16-hours following treatment. PBS was used as a negative control. **g** Percent wound healing quantification show Fontan post-op sEVs having a significantly higher percentage of wound closure when compared against Fontan pre-op sEVs. Glenn pre-op and post-op sEVs showed no significant differences in promoting wound closure. Graphed as mean ± SD. Significance was tested with a two-way repeated measure ANOVA with a Tukey posthoc. **h** Glenn and Fontan pathway analysis for serum and sEVs show an enrichment for vasculature development and tube morphogenesis in Fontan sEVs. ns = not significant, * *P* < 0.05. ** *P* < 0.01.

We next sought to identify differences in the transcriptome between serum and their sEVs postoperatively. After combining both our Fontan and Glenn datasets, our analysis revealed that 8967 RNA species were uniquely found in sEVs, 54 were found in the serum, and 476 were found in both (Fig. 4a, b). A comparative pathway analysis of differentially expressed genes in serum and sEV samples showed that the serum transcriptome is enriched for pathways involved in regulating RNA-protein complex assemblies and cytoplasmic translation. In contrast, sEV pathways are primarily enriched for extracellular matrix organization and circulatory system processes (Fig. 4c).

We then conducted a pathway interaction network analysis in our pre-op and post-op sEV samples. Pre-op sEVs showed a wider diversity of pathways ranging from cytoskeleton organization, ALK signaling, aerobic respiration, and apoptosis (Fig. 4d). Post-op sEVs, however, had more restricted pathways. Notably, post-op sEVs were enriched for motility-focused pathways including microtubule assembly, cytoskeleton regulation, collagen proteins, and angiogenesis (Fig. 4e). Our pathway analysis indicated an enrichment of motility and angiogenesis pathways in post-op sEVs, prompting us to validate these findings. A wound healing assay in HUVEC cells treated with pre-op and post-op sEVs revealed a significant improvement in wound healing (*p* < 0.01) after a 16-hour treatment of Fontan post-op sEVs compared to Fontan pre-op sEVs, with a mean wound closure of 21.1% ± 5.9% in pre-op Fontan sEVs and 35% ± 4.1% in post-op Fontan sEVs (Fig. 4f). Fontan post-op sEVs, however, narrowly did not meet the statistically significant threshold when compared to the negative control (*p* = 0.0555) (Fig. 4g). Interestingly, there was no significant wound healing improvement (*p* = 0.506) between Glenn pre-op and post-op sEVs, who had a mean wound closure of 29.4% ± 8.5% and 37.9% ± 8.6% for pre-op and post-op samples, respectively (Fig. 4g). To investigate this phenotypic difference, we performed a pathway analysis between post-op sEV and serum in Fontan and Glenn samples. Our analysis revealed that, although both sample groups had overlapping pathways, Fontan sEVs are more enriched for pathways involving angiogenesis – vasculature development and tube morphogenesis – than Glenn samples (Fig. 4h).

### Identification of key post-op sEV RNAs in enhancing wound healing

**Fig. 5:**
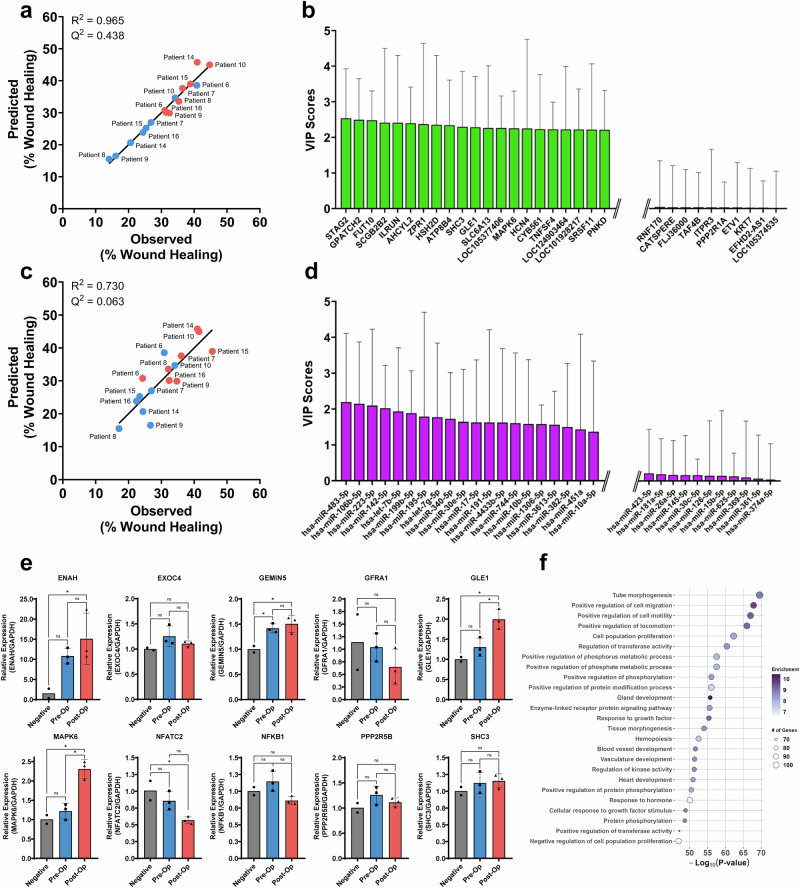
Identification of key sEV RNA cargo in promoting angiogenesis. **a** Observed vs predicted plot for the PLSR total RNA sEV model shows a strong correlation, indicating the model’s accuracy. **b** The top 15 and bottom 10 VIP gene scores for the total RNA-based PLSR model highlight the most influential genes, which may contribute significantly to the observed phenotypes. **c** Observed vs predicted plot from our PLSR miRNA sEV model similarly shows a strong correlation between our model and experimental results. **d** Top 15 and bottom 10 VIP scores reveal key miRNAs driving the predictive capability of our model. (**e**) RT-qPCR of ten VIP RNAs reveal MAPK6 and GLE1 as significantly (*p* < 0.01) upregulated following post-op sEV treatment implicating both genes in the development of collaterals. Graphed as mean relative expression ± SD. Significance was tested with a one-way repeated measure ANOVA with a Tukey posthoc. **f** Gene ontology analysis of target genes of the top VIP miRNAs shows enrichment in pathways related to cell migration, angiogenesis, and other regenerative processes. ns not significant, * *P* < 0.05. ** *P* < 0.01.

After having identified the pro-angiogenic phenotype in our sEVs, we next sought to pinpoint specific RNAs that may drive the observed response. First, we trained a PLSR model with both pre-op and post-op sEV total RNA to create a predictive model for our wound healing results. Our model accurately matched our experimental results (R^2^ = 0.965) but with a mild predictability score (Q^2^ = 0.438) (Fig. 5a). We next sought to improve the predictability of our model by re-training it and selecting the top 100 variables of importance for projection (VIPs) (Fig. 5b, Supplementary Table 5). Restricting our model to the top 100 VIPs significantly improved its predictability score (Q^2^ = 0.936) and better matched our experimental results (R^2^ = 0.997), suggesting that our selected VIP genes may be sufficient to build a robust PLSR model (Supplementary Fig. 3a-b). To further validate the contribution of selected VIP genes, we performed RT-qPCR to assess whether direct RNA transfer via pre-op and post-op Fontan sEVs would enhance or decrease the presence of VIP transcripts in endothelial cells. Among the genes tested, GLE1 (*p* < 0.05) and MAPK6 (*p* < 0.01) were significantly upregulated following post-op sEV treatment when compared to both pre-op and negative control (mean difference of –0.69 ± 0.18 and –1.09 ± 0.17 for GLE1 and MAPK6, respectively) (Fig. 5e). Additionally, genes such as ENAH and NFATC2 exhibited significant differences (*p* < 0.05) between post-op and the negative control, though no significant changes were observed between pre-op and post-op sEV treatments (Fig. 5e). To better elucidate the functional role of our VIP genes in the cell, we performed a protein-protein interaction network analysis using the STRING database^16^. Using our top 100 VIP genes as input, we identified shared protein interactions among them and selected for the top number of interactors under the premise that overlapping interactions would have magnified influence on cellular processes. Interestingly, our network revealed that MAPK6 and GLE1 had one of the fewest interactors while NFKB1 and GFRA1 had the highest number of protein interactors among all VIP genes. Conversely, CDH1, CTNNB1, and SRC had the highest number of VIP gene interactions (Supplementary Fig. 3e).

We next turned our attention to studying whether miRNAs contribute to the observed post-op sEV phenotypes. In parallel to our previous model, we built a PLSR model to identify key miRNA drivers in our sEVs. This initial model exhibited a low predictability score (Q^2^ = 0.063) but accurately matched our experimental results (R^2^ = 0.730) (Fig. 5c). Restricting the model to the top 50 miRNAs significantly improved its predictability (Q^2^ = 0.447) (Fig. 5d, Supplementary Fig. 3c, d). GO pathway analysis of the top 50 miRNAs revealed enrichment for angiogenesis-associated processes: tube morphogenesis, positive regulation of cell migration, positive regulation of cell motility, and positive regulation of phosphorylation (Fig. 5f). A full table of VIP miRNAs can be found on Supplementary Table 6. To assess the functional significance of our top VIP miRNAs in angiogenesis, we transfected miRNA inhibitors following sEV treatment. Out of our tested miRNAs, we observed that the inhibition of hsa-miR-340-5p significantly diminished the wound healing response in post-op sEVs (mean difference: –24.7 ± 9.4, *p* < 0.05) while inhibition of hsa-miR-199b-5p improved the wound healing response in pre-op sEVs (mean difference: 14.2 ± 5.1, *p* < 0.05) (Supplementary Fig. 4a, b).

### sEV tissue origin shifts following a Fontan or Glenn procedure

**Fig. 6:**
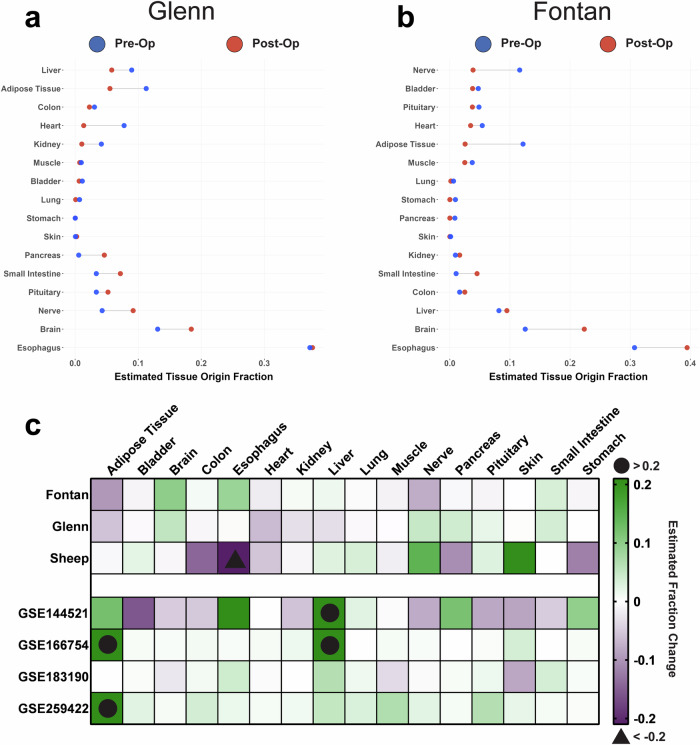
EV-origin deconvolution of Fontan and Glenn pre-op and post-op sEV samples. **a** Cleveland plot of Glenn sEVs reveals an increase in tissue fraction following surgery from nerve, brain, and pancreatic tissues while a decrease in cardiac sEVs occurs postoperatively. The difference in magnitude between pre-op (blue) and post-op (red) tissue fractions is designated via a connecting black line. X-axis represents the estimated fraction of sEVs that come from one of the 16 tissue types. **b** Cleveland plot of Fontan model sEVs similarly reveals an increase in tissue fraction following surgery from brain, esophagus, and hepatic tissues while a decrease in cardiac, nerve, and adipose sEVs occurs postoperatively. **c** Our study’s sEV tissue origin plotted against the tissue origins from a previously published sheep Fontan model and four publicly available datasets reveal that a decrease in cardiac sEVs and an increase in brain sEVs is unique to both the Fontan and Glenn procedures. Sheep subjects ranged between one to two years of age, an equivalent of early adolescence and early adulthood in humans.

Finally, after having pinpointed the role of specific sEV cargo in driving functional responses, we investigated whether the circulating sEV transcriptome had the potential to act as a robust tool to monitor tissue response and adaptation post-Fontan or Glenn surgery. We used the EV-Origin deconvolution algorithm to quantify the fraction of sEVs coming from 16 different tissue types^17^. EV-Origin deconvolution revealed a dramatic shift in sEV tissue origin following surgery. For Glenn samples, we observed an increase in estimated tissue origin fraction from nerve (0.05), brain (0.05), and pancreatic (0.04) tissues. Interestingly, we also observed a decrease in cardiac (0.06) fraction postoperatively (Fig. 6a). Fontan samples had relatively similar fraction patterns. Notably, we observed an increase in the origin fraction from the brain (0.10), esophagus (0.09), and a slight increase in hepatic fractions (0.01). Cardiac (0.02), nerve (0.08), and adipose (0.1) fractions significantly decreased following the Fontan surgery (Fig. 6b).

To strengthen our findings, we employed two validation strategies: first, we used EV-Origin to analyze sEV transcriptomes from our group’s previously published sheep Fontan animal model. Second, we utilized the datasets previously mined to control our differential gene expression analysis to evaluate if the tissue responses we observed were a result of a general surgical response rather than Fontan or Glenn-specific outcomes. Like our human samples, we observed a significant shift in sEV origin fraction on the sheep model following surgery. The fraction of sheep sEVs originating from nervous, hepatic, lung, and dermis tissues increased by 0.14, 0.04, 0.03, and 0.21, respectively. We also detected a decrease in tissue origin fraction of 0.06 from cardiac tissue, 0.12 from the stomach, 0.11 from the pancreas, and 0.14 from the colon (Fig. 6c).

A direct comparison of Fontan, Glenn, sheep, and other publicly available circulating sEV datasets revealed that some of our EV-Origin results are unique to the Fontan and Glenn procedures (Fig. 6c). Our results showed that the Fontan and Glenn procedure significantly decreases cardiac sEV fractions and increases sEVs originating from the brain. Interestingly, we observed that all datasets except for the Glenn showed a significant increase in hepatic sEVs.

## Discussion

To our knowledge, we report the first transcriptomic analysis of circulating RNA species in serum and serum-derived small extracellular vesicles (sEVs) throughout the perioperative period of the Fontan and Glenn procedures. This study aimed to identify transcriptomic changes in response to the Fontan and Glenn procedures, pinpoint viable circulating RNA-based biomarkers that could serve as predictive indicators of adverse outcomes in patients with the Fontan physiology and elucidate the roles of circulating sEVs as mediators in the development of systemic complications thereby enhancing our understanding of the underlying mechanisms and improving prognostic capabilities. Through this study, we gained valuable insight into the potential role of sEVs in promoting collateral development within the Fontan physiology, while identifying specific RNA cargo that could serve as biomarkers for this process.

We first chose to conduct an untargeted characterization of the serum and circulating sEV transcriptome. We gathered serum and circulating sEV samples from patients with a variety of single-ventricle defects before and after a Glenn or Fontan procedure. After isolation of these sEVs, we assessed their morphology using negative staining and nanoparticle tracking analysis. Neither method detected any meaningful change in sEV size or shape—an observation consistent with previous serum-derived sEV reports – with both methods reporting a globular sEV shape and a mean size range of around 100 nm^25,28^. Following sEV physical characterization, we compared the pre- and postoperative serum and sEV transcriptomes in Fontan and Glenn patients. Interestingly, we identified a substantial amount of RNAs differentially expressed in Fontan sEVs while all other samples showed almost no increase in post-op RNAs when compared to their pre-op counterparts. This implies a more significant systemic response to the Fontan hemodynamics than to the Glenn, which could underline the stronger sEV phenotypic outcomes seen in our in vitro assays. This systemic response was reinforced in our hierarchical clustering of sEVs and serum where we showed that the pre- and postoperative samples cluster separately from one another. In terms of random effects, no clustering patterns were detected based on a patient’s race and sex in Glenn samples; however, we could not clearly distinguish an effect of race in our Fontan samples as many samples came from a predominantly white patient population which limited our race-based clustering. Strikingly, we observed a better clustering pattern separating pre-op and post-op serum and sEV samples in heatmaps rather than the principal component analysis, suggesting that much of the biological differences between the two states lies in the top variable genes in the datasets. The overlap in pre-op and post-op principal component analysis groups can be due to technical or patient-to-patient differences, as shown in previous clinical reports^8^. These technical differences can be seen from our low alignment rates in some samples – especially in serum samples – which may have restricted our ability to completely capture the biological variability for each group. To be mindful of this, we employed a paired-matched design and normalized the library-size to account for differences in total reads and focused our study primarily on the more robust sEV transcriptome. Nonetheless, for future studies, greater sequencing depth through a larger cohort collection and volume of sEVs or serum RNAs will be required.

We have previously reported on the transcriptome differences between serum-derived sEVs and their respective serum in a sheep Fontan model^8^. The same was seen in our cohort where serum RNAs were predominately involved in regulating translation processes while pre-op and post-op sEV RNAs from both surgical groups appeared to be involved in pathways involved in regulating angiogenesis. Circulating sEVs have been shown to modulate angiogenesis, so we reasoned that a shift in the transcriptome profile – as observed in our pre-op and post-op sEVs—would invariably result in significant alterations in the capacity of these sEVs to modulate angiogenesis^29^. Additionally, many reports highlight the formation of venovenous collaterals following a Fontan procedure^9,30–32^. The formation of these structures is mechanically attributed to the balancing of the abnormally high central venous pressure caused by a patient’s remodeled single-ventricle physiology^32^. Our transcriptomics findings led us to hypothesize that post-op sEVs may act as mediators in collateral formation. However, it is well-established that chronic hypoxia promotes vessel growth via the secretion of pro-angiogenic factors, and thus, due to the perioperative nature of our experimental design, we could have been observing remnants of a chronic hypoxic sEV response in pre-Fontan physiology^9^. A scratch wound healing assay directly comparing pre-op and post-op sEVs revealed a statistically significant difference in wound healing between Fontan pre-op and post-op sEVs but not in Glenn samples, suggesting that there is further upregulation of pro-angiogenic cargo independent of chronic hypoxia in Fontan post-op sEVs. Pathway analysis supports our findings with more pro-angiogenic cargo in post-op Fontan sEVs than pre-op Fontan sEVs. While the *p*-value for post-op Fontan samples (*p* = 0.0555) was just above the threshold for significance when compared to negative control, we believe that the magnitude of the biological response supports our hypothesis. Nevertheless, additional sEV RNA data from a distant period after Fontan operation will be needed to validate our results. Aside from hypoxia, the activation of regenerative and healing mechanisms from a large post-operative wound may also be the impetus and origin for our observed phenotypes. A clear separation between a short-term regenerative response and long-term collateral formation via sEVs should also be the focus in future longitudinal studies.

While sequencing technologies allow for thorough profiling of circulating sEVs, pinpointing specific sEV cargo that best predicts our observed response would allow for quicker and more cost-efficient methods to monitor collateral formations. Our group has previously shown that computational modeling via partial least squares regression (PLSR) can accurately pinpoint specific sEV RNA species that are vital for promoting an observed phenotype^33^. Initially, we built a PLSR model using the complete total RNA and miRNA datasets for pre-op and post-op sEVs, however, while both models had high correlation scores to our experimental data, both models also presented with low predictability scores. We reasoned that such scores were a result of unnecessary noise feeding into our model, and by restricting the model to the top VIP RNAs or miRNAs, we observed much higher predictability scores. These more refined models indicate that the gene set critical for monitoring collateral formation may be significantly smaller than we previously anticipated. We tested our model by quantifying RNA transcripts following pre-op or post-op Fontan sEV treatment in endothelial cells. Out of all the tested RNAs, MAPK6 and GLE1 showed a significant upregulation following post-op sEV treatment. GLE1 is an RNA exporter with unclear roles in angiogenesis^34,35^. However, MAPK6 is a critical integration point for multiple signaling pathways, and its role in promoting angiogenesis is well-documented^36,37^. In addition to MAPK6 and GLE1, our inhibition experiments indicate that miR-199b-5p and miR-340-5p play opposing roles in promoting wound healing, with miR-199b-5p acting as an inhibitor and miR-340-5p as a stimulator. These findings align with existing literature and suggest that miR-199b-5p is upregulated in pre-op sEVs but decreases post-Fontan, allowing a shift toward a more pro-angiogenic profile^38,39^. miR-340-5p’s pro-angiogenic role has also been documented though it is important to note that our findings are based on an in vitro HUVEC model^40^. While this model provides valuable insights, it may not fully replicate the complexity of collateral formation in vivo. Further research should explore whether the up- or down-regulation of these transcripts in circulating sEVs persists in patients with developed collaterals and whether they can be used for diagnostic purposes, potentially incorporating more physiologically relevant models for validation.

Lastly, we wanted to determine whether circulating sEVs could also be used to monitor tissue responses induced by surgery and the unique physiology introduced by staged surgical palliation. A key aspect of sEVs is their unique property to reflect their parent cell via their cargo^6^. This allows sEVs to serve as non-invasive indicators of tissue activity, with their secretion levels potentially reflecting responses to the altered hemodynamics of the Fontan or Glenn procedures. We decided to use EV-Origin, a powerful deconvolution tool, to assess systemic tissue responses postoperatively^17^. Our EV-Origin analysis revealed that a decrease in cardiac sEVs and an increase in brain sEVs appear to be a distinct response to the Fontan or Glenn procedures. Interestingly, despite the sheep animal model being closer to the Fontan physiology, we were not able to detect a complete similarity in sEV origin between human and sheep Fontan samples, however, the decrease in cardiac sEV remains consistent for the sheep Fontan. The observed gradual decrease in cardiac sEVs is interesting and could be attributed to the more favorable conditions in the single ventricle, such as reduced volume load and improved oxygenation, that follow the Fontan procedure^4^. Similarly, the increase in brain sEVs coincides well with the known complications in the brain following the Fontan palliation^41^. Nevertheless, a more detailed study should be conducted by isolating and characterizing the transcriptome of these cardiac and brain sEVs. The isolation of these sEVs – via cardiac and brain specific sEV markers - would be an effective approach to further understand the systemic implications of this response. Further, more longitudinal studies will be needed in both human and animal models to better correlate the increased or decreased secretion of circulating sEVs and the physiological state of their tissue of origin.

In summary, our study revealed a clear transcriptomic difference between both Fontan and Glenn preoperative and postoperative sEV samples with milder differences found in the circulating serum transcriptome. Fontan post-op sEVs showed a stronger pro-angiogenic function when compared to sEVs originating from other groups, and we identified MAPK6, GLE1, hsa-miR-340-5p, and hsa-miR-199b-5p as key RNA drivers for the observed wound healing response. Further, sEV deconvolution revealed that cardiac and brain sEVs change in response to either Fontan or Glenn surgery in humans. The outcome of this study provides evidence for sEVs as potent biomarkers in the formation of venovenous collaterals and systemic patient monitoring. Lastly, our analysis proposes key RNAs and miRNAs that can be used as tools to be used in prognostic applications.

## Supplementary information

Supplementary Figures

## Data Availability

Sequencing data for this study can be found in the Gene Expression Omnibus with accession number: GSE269879. Data from this study is also available upon reasonable request.

## Code availability

Code used for this study can be requested from the corresponding author upon reasonable request.
