# Supplementary material for: Transcriptomic analysis of circulating extracellular vesicles during the perioperative period of Fontan and Glenn surgery
Source: NPJ Cardiovasc Health. 2024 Dec 18;1:36. doi: 10.1038/s44325-024-00039-1 (PMC12912405; doi:10.1038/s44325-024-00039-1)
Supplement: Supplementary file 1 — Supplementary Figures [file 44325_2024_39_MOESM1_ESM.pdf]

Supplementary Figures

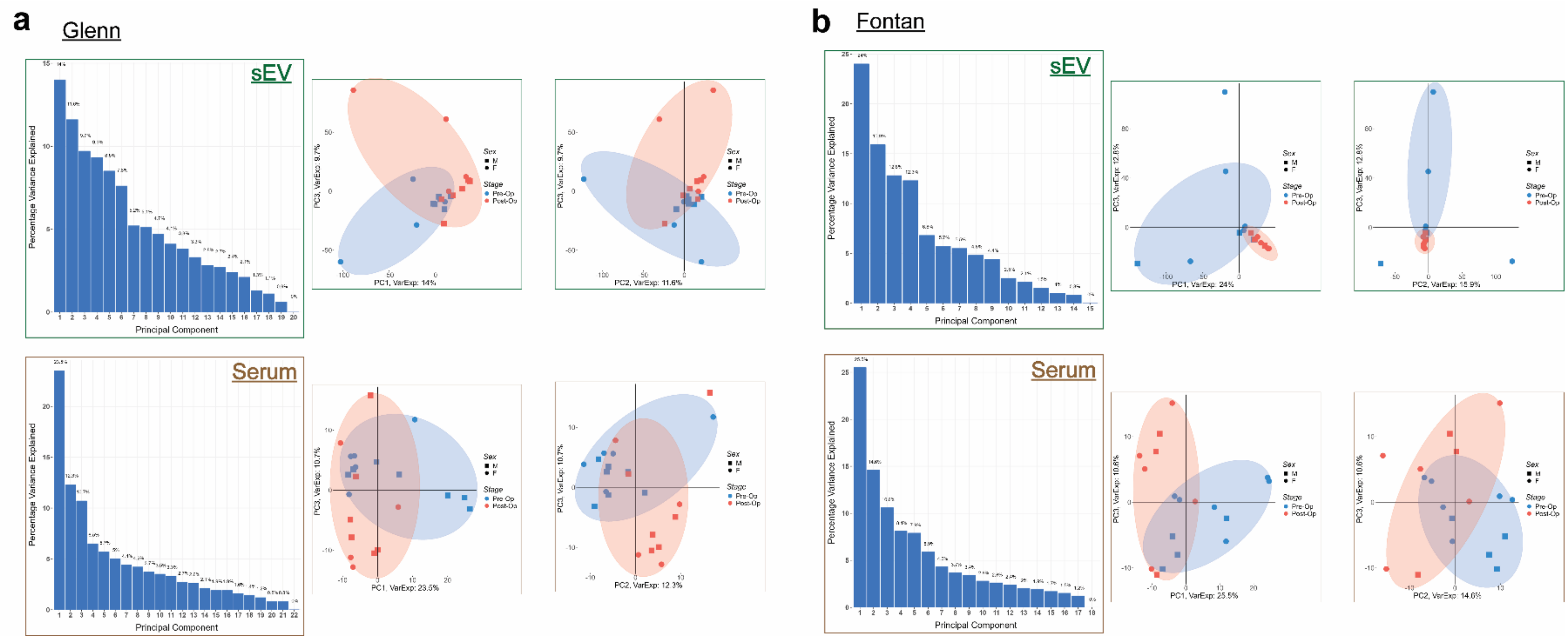

Supplementary Figure 1. Top principal components of sEV and serum across Fontan and Glenn patients. (**a**) Scree plot of Glenn sEVs and serum showing that the top six and top three components, respectively, account for the majority of variance in the datasets. Principal component analysis of the top three components reveals clustering based on pre-op and post-op samples. (**b**) Scree plot of Fontan samples also show that the top four and three principal components explain most of the variance in sEVs and serum, respectively. Principal component analysis shows more variability in Fontan sEV samples, but a clustering separation is still seen between sEVs and serum.

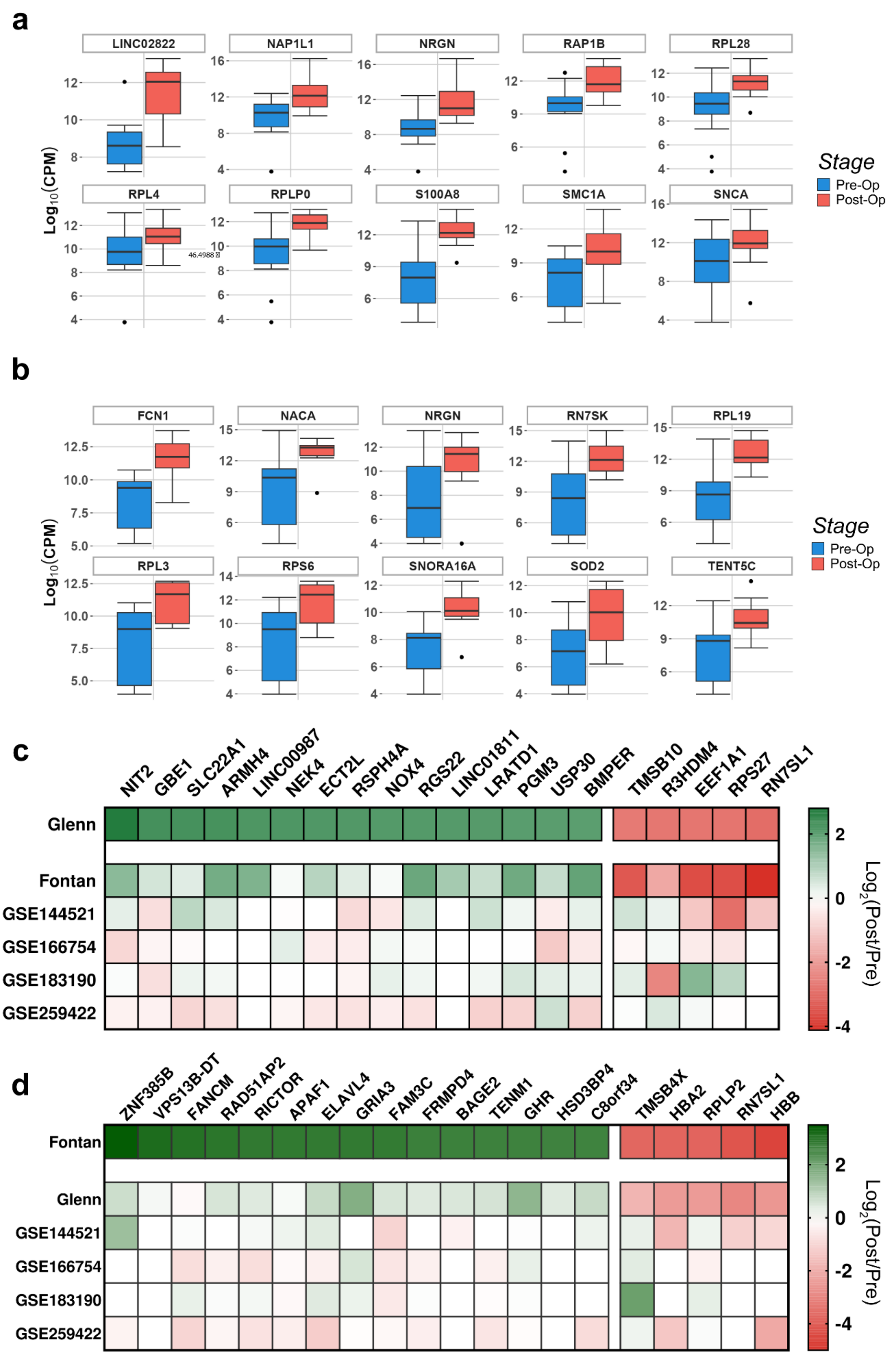
a
LINC02822
NAP1L1
NRGN
RAP1B
RPL28
RPL4
RPLP0
S100A8
SMC1A
SNCA
$Log_{10}(CPM)$
Stage
Pre-Op
Post-Op
b
FCN1
NACA
NRGN
RN7SK
RPL19
RPL3
RPS6
SNORA16A
SOD2
TENT5C
$Log_{10}(CPM)$
Stage
Pre-Op
Post-Op
c
NIT2
GBE1
SLC22A1
ARMH4
LINC00987
NEK4
ECT2L
RSPH4A
NOX4
RGS22
LINC01811
LRATD1
PGM3
USP30
BMPER
TMSB10
R3HDM4
EEF1A1
RPS27
RN7SL1
Glenn
Fontan
GSE144521
GSE166754
GSE183190
GSE259422
$Log_2(Post/Pre)$
2
0
-2
-4
d
ZNF385B
VPS13B-DT
FANCM
RAD51AP2
RICTOR
APAF1
ELAVL4
GRIA3
FAM3C
FRMPD4
BAGE2
TENM1
GHR
HSD3BP4
C8orf34
TMSB4X
HBA2
RPLP2
RN7SL1
HBB
Fontan
Glenn
GSE144521
GSE166754
GSE183190
GSE259422
$Log_2(Post/Pre)$
2
0
-2
-4

Supplementary Figure 2. Cross-validation of sEV transcriptomic analysis. (**a**) Boxplots of post-op Glenn serum's top ten differentially expressed genes. (**b**) Boxplots of post-op Fontan serum's top ten differentially expressed genes. (**c**) Heatmap cross-comparison between the top and bottom differentially expressed genes and four publicly available datasets reveal that Glenn genes are uniquely expressed following the procedure. (**d**) Heatmap cross-comparison between Fontan differentially expressed genes shows that they are distinctly expressed in our study.

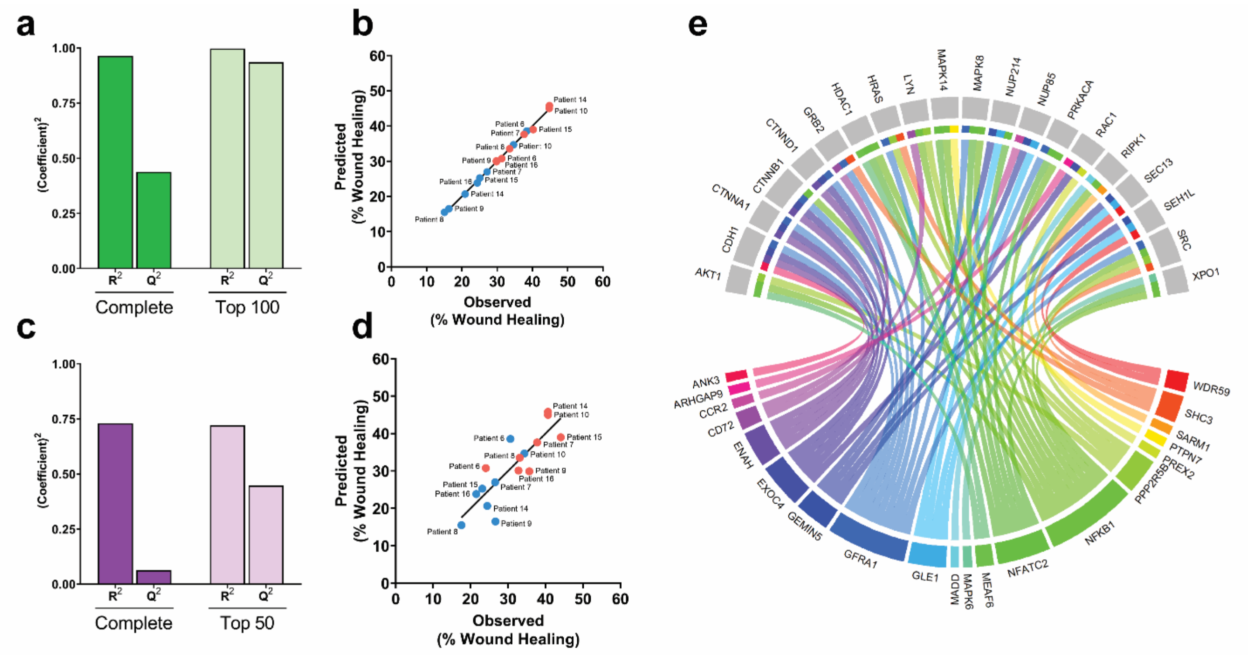

Supplementary Figure 3. PLSR refinement and protein-protein interaction network for top VIP RNAs. (**a**) Bar graph revealing that refinement of total RNA PLSR model to the top 100 RNAs improves predictability (Q^2) score. (**b**) Observed vs predicted plot of refined model reveals the improvement of the model. (**c**) Bar graph showing refined miRNA PLSR model by restricting the model to the top 50 VIP miRNAs. (**d**) Observed vs predicted plot of refined miRNA model reveals improvement in our model following refinement. (**e**) Protein-protein interaction network of top VIP RNAs reveals interactors that can play a key role in promoting angiogenic processes.

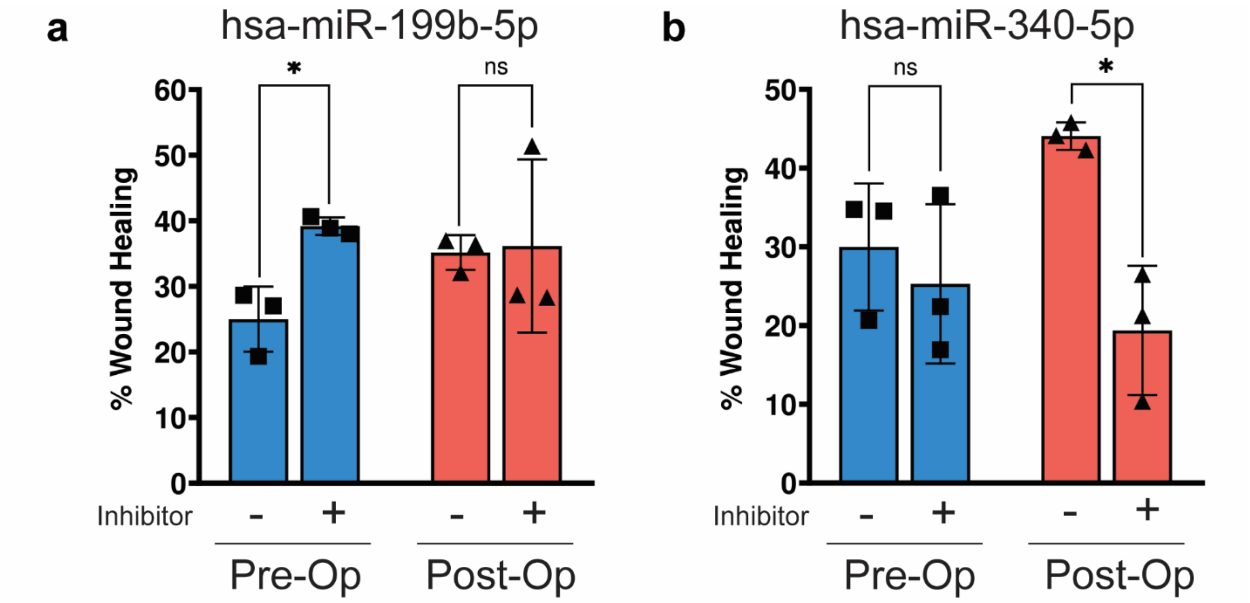

Supplementary Figure 4. Validation of predicted miRNA from PLSR model. (**a**) Wound healing assay results following inhibition of hsa-miR-199b-5p and pre- or post-op sEV treatment. Inhibition of hsa-miR-199b-5p in pre-op sEVs improves pre-op sEV pro-angiogenic function. (**b**) Wound healing assay results following inhibition of hsa-miR-340-5p and pre- or post-op sEV treatment. Inhibition of hsa-miR-340-5p in post-op sEVs attenuates their ability to stimulate wound closure.

Supplementary Table 1. Patient Characteristics

Patient Number	Pre-Op					Post-Op				
	Surgery	Sex	Race	Ethnicity	Age	Surgery	Sex	Race	Ethnicity	Age
**Patient 1**	Glenn	Male	Asian	Non-Hispanic or Non-Latino	5 months	Glenn	Male	Asian	Non-Hispanic or Non-Latino	5 months
**Patient 2**	Glenn	Male	White	Non-Hispanic or Non-Latino	3 months	Glenn	Male	White	Non-Hispanic or Non-Latino	3 months
**Patient 3**	Fontan	Female	White	Hispanic or Latino	5 years	Fontan	Female	White	Hispanic or Latino	5 years
**Patient 4**	Glenn	Female	Unknown	Hispanic or Latino	4 months	Glenn	Female	Unknown	Hispanic or Latino	4 months
**Patient 5**	Fontan	Male	White	Non-Hispanic or Non-Latino	3 years	Fontan	Male	White	Non-Hispanic or Non-Latino	3 years
**Patient 6**	Glenn	Female	White	Non-Hispanic or Non-Latino	4 months	Glenn	Female	White	Non-Hispanic or Non-Latino	4 months
**Patient 7**	Fontan	Female	White	Hispanic or Latino	3 years	Fontan	Female	White	Hispanic or Latino	3 years
**Patient 8**	Fontan	Female	White	Hispanic or Latino	4 years	Fontan	Female	White	Hispanic or Latino	4 years
**Patient 9**	Fontan	Female	White	Non-Hispanic or Non-Latino	2 years	Fontan	Female	White	Non-Hispanic or Non-Latino	2 years
**Patient 10**	Glenn	Male	Black	Non-Hispanic or Non-Latino	5 months	Glenn	Male	Black	Non-Hispanic or Non-Latino	5 months
**Patient 11**	Fontan	Male	White	Non-Hispanic or Non-Latino	3 years	Fontan	Male	White	Non-Hispanic or Non-Latino	3 years
**Patient 12**	Glenn	Male	White	Hispanic or Latino	5 months	Glenn	Male	White	Hispanic or Latino	5 months
**Patient 13**	Glenn	Male	White	Hispanic or Latino	4 months	Glenn	Male	White	Hispanic or Latino	4 months
**Patient 14**	Glenn	Male	White	Non-Hispanic or Non-Latino	4 months	Glenn	Male	White	Non-Hispanic or Non-Latino	4 months
**Patient 15**	Fontan	Male	White	Non-Hispanic or Non-Latino	6 years	Fontan	Male	White	Non-Hispanic or Non-Latino	6 years
**Patient 16**	Glenn	Female	Black	Non-Hispanic or Non-Latino	4 months	Glenn	Female	Black	Non-Hispanic or Non-Latino	4 months
**Patient 17**	Glenn	Female	White	Hispanic or Latino	6 months	Glenn	Female	White	Hispanic or Latino	6 months
**Patient 18**	Fontan	Female	White	Non-Hispanic or Non-Latino	4 years	Fontan	Female	White	Non-Hispanic or Non-Latino	4 years
**Patient 19**	Glenn	Male	Black	Non-Hispanic or Non-Latino	4 months	Glenn	Male	Black	Non-Hispanic or Non-Latino	4 months
**Patient 20**	Fontan	Female	Black	Non-Hispanic or Non-Latino	5 years	Fontan	Female	Black	Non-Hispanic or Non-Latino	5 years
**Patient 21**	Glenn	Female	White	Non-Hispanic or Non-Latino	6 months	Glenn	Female	White	Non-Hispanic or Non-Latino	6 months
**Patient 22**	Fontan	Male	White	Non-Hispanic or Non-Latino	3 years	Fontan	Male	White	Non-Hispanic or Non-Latino	3 years
**Patient 24**	Fontan	Female	White	Non-Hispanic or Non-Latino	4 years	Fontan	Female	White	Non-Hispanic or Non-Latino	4 years
**Patient 25**	Fontan	Female	Black & White	Unknown	5 years	Fontan	Female	Black & White	Unknown	5 years
**Patient 28**	Fontan	Female	White	Unknown	4 years	Fontan	Female	White	Unknown	4 years

Supplementary Table 2. Patient Diagnosis

Patient Number	Diagnosis
**Patient 1**	Heterotaxy syndrome, right atrial isomerism, complete atrioventricular canal defects, DORV, PA band and PDA ligation, severely hypoplastic left ventricle
**Patient 2**	DILV, L-TGA, pulmonary atresia, VSD, EAT. S/P 3.5 modified BTT shunt, atrial septectomy, and PDA ligation, ECMO
**Patient 3**	Unknown
**Patient 4**	Single ventricle DILV, VSD
**Patient 5**	DORV, D-TGA, VSD, PS, s/p Glenn
**Patient 6**	DILV, D-TGA, Hypoplastic RV, VSD, S/P pulmonary artery bands
**Patient 7**	unbalanced AVC, D-TGA, pulmonary atresia, and heterotaxy
**Patient 8**	Functionally univentricular heart; Double inlet right ventricle; Severe vavlar and subvalvar aortic stenosis; Coarctation of the aorta.
**Patient 9**	Heterotaxy syndrome (LAI), unbalanced AV canal (RV dominant), DORV with uncommitted VSD, interrupted IVC, multiple muscular VSDs
**Patient 10**	PA/IVS s/p PDA stent x2. hypoplastic right heart syndrome, right coronary sinusoids s/p ductal stenting
**Patient 11**	Single ventricle with unbalanced atrioventricular canal
**Patient 12**	DORV with no subaortic or subpulmonary obstruction, unbalanced AVC defect with RV dominance, and severe LV hypoplasia
**Patient 13**	L-TGA, severe pulmonary stenosis, ventriculo-arterial discordance and atrioventricular discordance, large VSD
**Patient 14**	DORV, VSD, coarctation of the aorta, hypoplastic arch, and subaortic stenosis
**Patient 15**	DILV, hypoplastic RV, LTGA, hypoplastic aortic arch, CoA
**Patient 16**	PA/IVS, confluent branch PA's, hypoplastic TV and right CA to RV sinusoid
**Patient 17**	HLHS MA/AA with mild aortic valve regurgitation
**Patient 18**	Double inlet left ventricle, normally related great vessels, mild pulmonary valve stenosis s/p bidirectional Glenn
**Patient 19**	HLHS (MS/AS)
**Patient 20**	HLHS (MA/AA)
**Patient 21**	PA/IVS, restrictive atrial septum, double outlet RA, RC origin atresia, and large RV to CA fistulae
**Patient 22**	HLHS
**Patient 24**	Tricuspid Atresia
**Patient 25**	Heterotaxy, RAI, PA, VSD, partial AV canal, and left posterior MCA infarct
**Patient 28**	Heterotaxy, Dextrocardia, DILV/DORV

Supplementary Table 3. EV Total RNA sequencing alignment metrics. Pre-Op = preoperative. Post-Op = postoperative.

Sample Name	Percent Reads Aligned	Total Sequenced Reads (Millions)
Patient 1 Pre-Op	19.80%	38.8
Patient 2 Pre-Op	14.70%	36.8
Patient 3 Pre-Op	5.30%	42.5
Patient 4 Pre-Op	5.90%	52.3
Patient 5 Pre-Op	18.50%	42.7
Patient 6 Pre-Op	6.20%	33.2
Patient 7 Pre-Op	5.70%	40.2
Patient 8 Pre-Op	9.80%	44.9
Patient 9 Pre-Op	27.30%	34.2
Patient 10 Pre-Op	5.70%	39.3
Patient 12 Pre-Op	40.80%	35.3
Patient 14 Pre-Op	26.10%	37
Patient 15 Pre-Op	28.50%	35.1
Patient 16 Pre-Op	33.50%	35.1
Patient 17 Pre-Op	4.80%	37.4
Patient 18 Pre-Op	20.30%	35.3
Patient 1 Post-Op	47.60%	33.7
Patient 2 Post-Op	84.40%	38
Patient 3 Post-Op	85.00%	50.8
Patient 4 Post-Op	63.70%	36.3

Patient 5 Post-Op	29.70%	34.4
Patient 6 Post-Op	5.60%	35.2
Patient 7 Post-Op	50.00%	44.9
Patient 8 Post-Op	80.90%	38.6
Patient 9 Post-Op	72.70%	43.6
Patient 10 Post-Op	38.20%	38.9
Patient 11 Post-Op	50.30%	34.3
Patient 12 Post-Op	31.10%	32.5
Patient 13 Post-Op	18.70%	38
Patient 14 Post-Op	85.90%	45.9
Patient 15 Post-Op	88.20%	39.2
Patient 16 Post-Op	79.70%	33.3
Patient 17 Post-Op	20.00%	45.6

Supplementary Table 4. Serum Total RNA sequencing alignment metrics. Pre-Op = preoperative. Post-Op = postoperative.

Sample Name	Percent Reads Aligned	Total Sequenced Reads (Millions)
Patient 1 Pre-Op	4.10%	52.7
Patient 2 Pre-Op	4.80%	57.3
Patient 3 Pre-Op	3.00%	43.4
Patient 4 Pre-Op	13.20%	49.4
Patient 5 Pre-Op	7.80%	62.5
Patient 6 Pre-Op	4.10%	33.5
Patient 7 Pre-Op	74.30%	47
Patient 8 Pre-Op	17.10%	36.8
Patient 9 Pre-Op	66.40%	48.4
Patient 10 Pre-Op	80.80%	54.5
Patient 11 Pre-Op	19.00%	48.1
Patient 12 Pre-Op	77.20%	63.7
Patient 13 Pre-Op	29.30%	49.7
Patient 14 Pre-Op	56.10%	32
Patient 15 Pre-Op	4.30%	26
Patient 16 Pre-Op	6.50%	20.5
Patient 17 Pre-Op	5.50%	24.8
Patient 18 Pre-Op	7.80%	41.7
Patient 19 Pre-Op	19.20%	28.4
Patient 20 Pre-Op	22.90%	56.5

Patient 21 Pre-Op	43.50%	49.8
Patient 22 Pre-Op	4.70%	29.1
Patient 1 Post-Op	12.50%	28.8
Patient 3 Post-Op	26.60%	37.2
Patient 5 Post-Op	10.10%	25
Patient 6 Post-Op	9.90%	24
Patient 7 Post-Op	19.90%	42.8
Patient 8 Post-Op	1.00%	47.7
Patient 9 Post-Op	1.30%	45
Patient 10 Post-Op	17.70%	35
Patient 11 Post-Op	1.30%	0.9
Patient 12 Post-Op	6.00%	59.9
Patient 13 Post-Op	1.50%	43.9
Patient 14 Post-Op	1.80%	57.3
Patient 16 Post-Op	14.60%	43.1
Patient 17 Post-Op	3.20%	69.3
Patient 18 Post-Op	0.90%	57.5
Patient 19 Post-Op	5.80%	60.1
Patient 21 Post-Op	1.40%	64.9

Patient 22 Post-Op	2.20%	0.5

Supplementary Table 5. Top 1000 Total RNA PLSR VIP Scores

Primary ID	VIP Score	VIP ± Standard Error
STAG2	2.534300089	1.391080022
GPATCH2	2.493910074	1.15436995
FUT10	2.480900049	0.824980974
SCGB2B2	2.412630081	2.089900017
ILRUN	2.411789894	1.889050007
AHCYL2	2.397880077	1.012940049
ZPR1	2.37210989	2.267570019
HSH2D	2.35187006	1.949339986
ATP8B4	2.343879938	1.265519977
SHC3	2.296459913	1.561300039
GLE1	2.284549952	1.427729964
SLC6A13	2.263819933	1.745499969
LOC105377406	2.263710022	0.898850977
MAPK6	2.252949953	1.048300028
HCN4	2.250509977	2.503989935
CYB561	2.230489969	1.535179973
TNFSF4	2.225349903	0.766929984
LOC124903464	2.223140001	1.770769954
LOC101928217	2.222110033	1.137439966
SRSF11	2.218519926	1.850849986
PNKD	2.212369919	1.107030034

ZNF717	2.209899902	1.680609941
LOC124902750	2.207809925	1.168169975
PREX2	2.19385004	1.37469995
NFATC2	2.177390099	0.996378005
DYM	2.173949957	1.716969967
ARHGAP9	2.170779943	2.534759998
NFKB1	2.165220022	1.567469954
LOC124901687	2.161170006	1.909649968
ANKRD36C	2.159320116	1.696390033
ZNF514	2.156239986	2.281339884
ABCC5	2.152620077	1.286200047
NUDC	2.15128994	1.236559987
LOC107986478	2.15083003	2.767990112
DCDC2C	2.14338994	1.541579962
LOC105376426	2.143049955	0.823463023
XRCC1	2.142329931	0.924413979
LOC101928361	2.141489983	1.21310997
SIK3	2.141400099	1.723070025
LOC124904701	2.138459921	1.887420058
MYO5B	2.137799978	1.303069949
GOSR2	2.13567996	1.48672998
PDCD10	2.135020018	1.455669999
ATP1A4	2.129970074	2.047060013

MEAF6	2.129859924	1.10868001
TBC1D20	2.125760078	0.713908017
PPP2R5B	2.123349905	1.398110032
DNAH10	2.11619997	2.517019987
GARS1-DT	2.1161201	1.490260005
ARMH4	2.114919901	1.686450005
GFRA1	2.113460064	1.305369973
CCDC80	2.113349915	1.038969994
CCDC27	2.111129999	1.702399969
PTPN7	2.109070063	1.244519949
CCR2	2.102760077	1.922189951
LOC112267955	2.102570057	1.039510012
MORC2	2.088089943	1.123520017
ARPC5	2.087559938	1.836799979
LOC124905096	2.082129955	2.605020046
SLC44A1	2.081140041	1.380460024
ZNF827	2.08100009	1.019860029
DGCR2	2.069350004	1.038570046
TANGO2	2.063730001	1.456019998
HEATR5A	2.063719988	1.505190015
NGDN	2.062000036	1.155230045
COL14A1	2.059250116	1.726250052
IDS	2.05704999	1.116219997

GEMIN5	2.055500031	1.799790025
VSIG2	2.052129984	1.183220029
RBM3	2.050189972	1.632130027
CLPB	2.049590111	1.14470005
CARD8	2.048290014	1.789190054
PCNX1	2.046629906	1.340610027
TMC5	2.045919895	1.80219996
CD72	2.044859886	1.418439984
CROCC2	2.040519953	1.612709999
TP53INP1	2.039129972	1.114410043
ENO1	2.038500071	0.955102026
NEO1	2.038409948	1.528380036
CDKAL1	2.036550045	1.216339946
ATG4B	2.035330057	2.247319937
EXOC4	2.022759914	1.098119974
AK9	2.022109985	1.358819962
ENAH	2.020469904	0.901669979
SARM1	2.019249916	1.743690014
SMIM12	2.01473999	1.835680008
ZNF746	2.014199972	2.730489969
PLXNC1	2.011780024	1.141939998
RALGAPA1	2.009360075	0.767885029
MADD	2.007499933	1.750970006

MEX3B	2.00744009	1.070979953
ANK3	2.007250071	1.051149964
SPATA24	2.005160093	1.406380057
CPVL	2.002929926	1.34758997
CNN3	2.000250101	2.273180008
SLC15A2	1.999029994	1.481930017
NDUFV3	1.997939944	1.624670029
WDR59	1.996850014	1.290179968
C1QTNF1	1.996680021	1.030969977
ERG28	1.996369958	2.029799938
LPGAT1	1.995859981	1.005789995
TRANK1	1.992879987	1.602159977
SCUBE2	1.992020011	2.224639893
LINC02923	1.99119997	1.929080009
POLH	1.990800023	1.420590043
FRY	1.990589976	1.528290033
PGAP4	1.987040043	1.639660001
LOC101927764	1.986289978	1.741140008
HNRNPA1	1.984660029	1.647169948
SOS2	1.98063004	1.806499958
LOC101928419	1.978860021	1.341809988
CABP1	1.978649974	2.327029943
LOC124904869	1.977900028	2.177020073

ARHGEF40	1.976300001	1.046659946
TTBK2	1.97439003	1.306339979
PWWP2A	1.974060059	2.014019966
METTL13	1.973760009	0.884845018
SERPINB1	1.972859979	2.121150017
ABTB2	1.971650004	2.03392005
SHMT2	1.969200015	1.491440058
PKNOX2	1.969130039	1.539890051
ZNF3	1.968219995	1.97250998
LOC107985900	1.967000008	1.156380057
UNC45B	1.966539979	2.568269968
LOC124904196	1.966510057	1.368510008
FN1	1.965610027	1.75406003
LINC01544	1.965010047	0.667667985
FRRS1	1.964419961	2.433459997
CHST9	1.963369966	1.622679949
RILPL2	1.963350058	1.611940026
UTP15	1.961099982	1.036219954
HSPBAP1	1.96081996	1.173290014
STX3	1.959699988	2.013050079
DNM3	1.959110022	1.179020047
PHKA2	1.955410004	1.250740051
EFCAB5	1.953870058	1.17098999

LOC101928202	1.952909946	0.699230015
ASIC3	1.951650023	2.489389896
KIAA0825	1.947239995	1.314970016
FBXO9	1.93671	1.598340034
ADRB1	1.936619997	0.861967027
PEA15	1.935119987	2.147130013
PTPRQ	1.933120012	1.325860023
SHISA7	1.92888999	1.040230036
NONO	1.928419948	1.307829976
ARF3	1.927379966	1.716650009
ARHGAP4	1.927299976	1.010439992
STIL	1.923740029	1.75296998
RPS15	1.922019958	0.711372972
TCERG1	1.919430017	1.614719987
WNT9A	1.919209957	1.513520002
CTNS	1.918079972	2.229890108
TCEA1	1.916710019	1.415639997
CDH26	1.91663003	1.063560009
TANGO6	1.916350007	2.085160017
GATA2	1.916350007	0.886820972
MICAL2	1.914739966	1.165660024
MLF1	1.912739992	2.158829927
LOC101928331	1.912330031	1.488430023

ABCA9	1.910789967	2.25999999
CLMN	1.910470009	1.069159985
LOC128966704	1.907969952	1.836969972
ZBTB7A	1.906649947	1.28113997
WDR64	1.906200051	1.80953002
LOC124903730	1.904489994	1.709499955
ISCU	1.904119968	1.208899975
LOC124903541	1.902400017	1.820590019
LATS2	1.902029991	1.541910052
TP63	1.900650024	1.953999996
ELK1	1.899569988	1.677999973
USP49	1.898810029	1.804219961
ASB4	1.898309946	1.956500053
ANP32A	1.897289991	0.838558018
OTULIN	1.897210002	1.168980002
SLC22A10	1.896039963	1.326580048
SPDL1	1.892040014	1.570000052
DTX3L	1.891849995	1.918239951
ADGRB3	1.891360044	2.238919973
LOC105376177	1.890959978	2.122230053
STK11IP	1.890259981	1.955330014
SPATA31E1	1.889400005	1.220800042
NOTCH2	1.886489987	1.812029958

FER1L6	1.886279941	1.666200042
RPS27	1.885959983	0.945999026
PAG1	1.885669947	0.821632028
CDK5R2	1.883820057	1.093760014
LOC107986350	1.881620049	1.792610049
MYMX	1.879930019	2.026350021
HNRNPD	1.879279971	1.562209964
HADHA	1.87804997	2.15102005
LSM3	1.877509952	1.678239942
MTIF3	1.877349973	1.916749954
LOC105374810	1.877110004	1.93543005
PTPN3	1.876369953	1.538120031
RPS4X	1.876270056	0.932694972
GPATCH2L	1.876150012	0.719847023
RCOR3	1.874609947	1.885750055
A2ML1	1.87421	0.629969001
PSEN1	1.873209953	1.655069947
HBS1L	1.873200059	1.6329
POC1A	1.871760011	1.826840043
LOC124905095	1.870890021	1.499279976
GORASP1	1.870159984	1.273859978
DPP4	1.870129943	2.246239901
MON1B	1.869609952	1.406630039

LOC124904458	1.869590044	1.620249987
DAZAP2	1.869519949	1.297389984
GALC	1.868630052	0.927268982
JAKMIP3-AS1	1.868280053	1.295359969
LAMP1	1.868229985	1.300819993
EXOC7	1.867259979	1.477349997
LGMN	1.865339994	2.170609951
CORO1C	1.864940047	0.81339699
FAM131B	1.863549948	1.707059979
RPA1	1.86255002	0.753234029
EMC8	1.862259984	1.683879972
CHEK1	1.86164999	1.678460002
BIN1	1.861549973	0.853730977
LOC105377744	1.859910011	1.170680046
LOC102725082	1.859670043	1.065440059
WDR31	1.859089971	1.931820035
SOCS6	1.85698998	1.300269961
SSRP1	1.856269956	1.893229961
DNAJC3	1.855290055	1.574419975
ANGPTL2	1.854969978	1.816540003
MSH2	1.854439974	1.209810019
RAD50	1.85442996	1.119330049
GPR141	1.853970051	1.623729944

LOC124902475	1.85323	1.084220052
CAND2	1.85315001	1.648010015
MYH16	1.852030039	2.762989998
LOC105375724	1.851799965	1.342700005
PIK3CB	1.850810051	1.591249943
DEFB121	1.84987998	1.101590037
KLHL29	1.849300027	2.316659927
ATP8A2	1.846060038	1.273579955
GABRD	1.844099998	1.824810028
LASP1	1.843500018	1.675320029
CAMK2B	1.842540026	1.926450014
CAT	1.84197998	1.165220022
GALK2	1.838400006	1.615059972
DESI2	1.837610006	0.59048301
LOC105377989	1.836120009	1.915030003
HOMER2	1.835950017	1.563660026
LOC124902644	1.83525002	1.064470053
H1-4	1.835170031	1.792940021
HOXB7	1.834720016	1.144580007
APAF1	1.834360003	1.49703002
A4GALT	1.834200025	1.591259956
RETREG3	1.832059979	1.214810014
NRDC	1.830970049	2.570739985

MYO16	1.829929948	2.033659935
SPHKAP	1.829390049	1.36243999
WIZ	1.829190016	1.258380055
LOC124902152	1.827209949	1.720989943
MEG3	1.826269984	2.048969984
RECQL5	1.825799942	2.550499916
MMAA	1.825539947	2.03647995
SERBP1	1.82494998	0.842463017
LOC105370168	1.823889971	1.472129941
OAS2	1.823709965	0.597548008
JARID2	1.82336998	1.293660045
RPS16	1.822870016	0.729470015
CDH22	1.819499969	1.842290044
ADAMTS4	1.817680001	1.92426002
LOC105374947	1.817299962	1.362679958
HNRNPM	1.817299962	0.989470005
AMZ1	1.816969991	2.478879929
NSUN2	1.816269994	1.470049977
LOC105376384	1.815790057	0.538447022
LOC107985957	1.815420032	1.230469942
RHOH	1.813159943	0.857222021
DLG2	1.812019944	1.088660002
IRX1	1.811439991	1.445670009

CDK11B	1.810889959	0.96648401
CSRP3-AS1	1.810559988	2.218189955
NOS3	1.810369968	2.172990084
DEK	1.809769988	1.02500999
PTGES3	1.809659958	1.244339943
SCN8A	1.809249997	1.076239944
RPE65	1.808200002	0.650529027
DGKI	1.80708003	1.116340041
LOC124903734	1.806550026	0.89784199
LOC105376266	1.805699944	1.441030025
LOC105369309	1.804949999	1.873749971
PRKCE	1.803519964	0.959384978
PCF11	1.803460002	1.933269978
PRNCR1	1.80298996	1.972350001
ANXA4	1.802850008	0.828167021
MGAT4C	1.802049994	2.256230116
NOX5	1.801679969	2.379869938
DENND2C	1.800799966	1.639369965
DNAJA4	1.800570011	1.212380052
ARL8B	1.800369978	1.557289958
TMEM201	1.798529983	1.586560011
ZC3H10	1.798099995	0.852265
ZNF221	1.797809958	2.088860035

TACC1	1.796270013	0.826882005
CNTNAP5	1.795959949	1.739869952
MPHOSPH8	1.795779943	2.189500093
SUZ12	1.795420051	2.396189928
IFT172	1.794950008	2.660670042
ROR2	1.794499993	1.302070022
LYPLAL1	1.794149995	0.768736005
NME6	1.792260051	1.32167995
SHISA9	1.792000055	1.993100047
LOC105375321	1.791419983	1.558339953
PDLIM7	1.791339993	1.116119981
ROS1	1.791090012	1.204210043
COTL1	1.790459991	0.859413028
LRP1B	1.788619995	1.532780051
PDE4D	1.787989974	0.996479988
SGMS1	1.785629988	0.886232018
CDADC1	1.785030007	2.012550116
HNRNPAB	1.784739971	1.733700037
TJP2	1.784039974	1.404809952
ABCC3	1.784019947	1.324130058
SHROOM4	1.783890009	1.189170003
RPS27A	1.78354001	1.09612
NRP2	1.782580018	1.431630015

TMEM39B	1.78234005	1.689429998
LOC107985714	1.780210018	1.248250008
ZNF488	1.778939962	2.37142992
CD74	1.778710008	0.661005974
LINC02718	1.776720047	2.072410107
STK38	1.776360035	0.806824982
LOC105375508	1.776329994	0.899345994
FGD1	1.775279999	1.339699984
PML	1.774749994	1.765519977
NBPF11	1.773360014	2.050199986
C8orf34	1.773249984	1.567499995
BCAR3	1.773139954	0.791483998
ATP6V0A2	1.772300005	1.596150041
BNC2	1.771970034	1.422809958
CDAN1	1.771970034	1.945070028
IKBKE	1.771299958	1.504130006
SLC39A9	1.769119978	0.794737995
DOCK1	1.768980026	0.688199997
FRMD8	1.768669963	1.868139982
MYH3	1.768569946	1.308879972
CPM	1.767650008	1.919399977
ERICH6B	1.767480016	1.300559998
FBN2	1.76662004	1.371770024

FANCE	1.765239954	1.842100024
CDX2	1.765059948	1.359300017
SIRPB1	1.764619946	1.439620018
PSMD4	1.764299989	1.748250008
LINC03025	1.76364994	1.057960033
RTCB	1.763200045	1.102820039
ELAVL2	1.762959957	2.690520048
UBE2V2	1.760810018	0.848030984
LOC105375655	1.760740042	0.898934007
AP4B1	1.759940028	1.250579953
EPHB1	1.759780049	2.190779924
SLC35D1	1.759490013	0.817627013
ZNF790	1.759070039	1.431849957
SLC14A2	1.758069992	1.784649968
UBE2O	1.756719947	0.609979987
NSUN6	1.756340027	0.881563008
ALG12	1.755480051	1.078089952
SAMD12	1.754649997	1.129959941
SYNCRIP	1.754570007	2.247200012
SLC38A5	1.754099965	1.959069967
MYL12A	1.754009962	1.659029961
ADRA1B	1.75395	1.249279976
ACSM3	1.752900004	2.123080015

VCAN	1.75177002	0.980592012
LOC124902386	1.748260021	1.645830035
ECEL1	1.748200059	1.406649947
CRELD1	1.748100042	1.594980001
LOC107985962	1.747689962	0.823553979
LOC124900234	1.746719956	1.722599983
PRRT4	1.745669961	0.884585023
HOXB3	1.745300055	1.828230023
DZIP1L	1.744019985	1.471099973
LINC00461	1.743589997	1.800430059
RPL26	1.743350029	1.108999968
SGK1	1.743109941	1.45381999
RBFOX1	1.742280006	1.5546
EPHB4	1.742220044	1.079450011
LOC729218	1.74090004	2.020060062
GNAI2	1.739850044	1.201140046
ARF6	1.739750028	0.859432995
LOC100506551	1.739439964	0.868734002
RPS19	1.738399982	1.369609952
ARHGAP42	1.738299966	1.514799953
LINC00482	1.737769961	0.765439987
PLBD2	1.737769961	2.387840033
SLC27A1	1.736979961	0.756763995

FGD5P1	1.73695004	1.823410034
ARRDC3	1.736670017	1.358940005
PPM1G	1.73664999	1.139770031
LOC124902269	1.736369967	2.165889978
ZSCAN12	1.735929966	2.125289917
AJAP1	1.735689998	0.893817008
MMP13	1.735620022	0.797029972
RRP1B	1.735520005	1.665449977
PRSS3	1.735159993	2.21329999
ERAP1	1.734799981	1.861819983
LOC284600	1.734259963	1.436740041
PLIN1	1.733659983	1.822399974
TUBGCP2	1.733180046	0.987713993
GABRA2	1.733070016	1.243170023
CCN4	1.732900023	1.38349998
P3H3	1.732710004	1.637240052
PPM1F-AS1	1.732480049	1.20618999
PRRC2B	1.732360005	0.758985996
LOC105377113	1.732079983	1.199419975
CUL4A	1.731639981	1.520409942
TMEFF2	1.731150031	1.685879946
LOC105375825	1.728430033	1.566580057
EIF3L	1.728330016	1.316470027

B4GALT6	1.728019953	2.227370024
LOC107985467	1.727689981	0.999938011
EIF2S2	1.727090001	1.48277998
PATL1	1.72578001	1.115720034
STXBP4	1.724519968	0.754621029
ANP32B	1.724300027	0.994461
STK36	1.723919988	0.973667979
EMC3	1.723860025	1.058529973
PODXL	1.723649979	2.909280062
TRPM3	1.723350048	2.00861001
BEND6	1.723099947	1.725649953
CRACR2B	1.723080039	1.058300018
PLCL1	1.720770001	2.31814003
SMYD1	1.720569968	2.320940018
PDE8A	1.719930053	2.266170025
GLB1L3	1.719820023	1.651110053
ST6GAL2	1.719640017	1.129369974
ABCG2	1.718709946	1.904739976
LOC105370462	1.717610002	1.178110003
H2BC4	1.717479944	0.695688009
BRPF3	1.717339993	0.927540004
ASL	1.717100024	0.837471008
KLB	1.716210008	1.228049994

DMXL2	1.715980053	0.753183007
HSPG2	1.71570003	2.663239956
RGS6	1.71528995	1.723950028
CEP63	1.713000059	1.601999998
ATXN1L	1.712890029	1.292000055
LOC105371953	1.711699963	1.308099985
TMSB10	1.710520029	0.920385003
ANKRD33B	1.710039973	1.755139947
RPS7	1.709779978	0.783810973
LOC124901978	1.709149957	1.033630013
ACP7	1.708799958	1.282400012
CDC25B	1.708299994	1.530369997
PPM1B	1.707759976	0.734448016
PFKFB2	1.706429958	1.674909949
HELZ2	1.706320047	1.282649994
LOC105370108	1.706230044	0.798386991
KIAA1328	1.705669999	1.160490036
LOC124900275	1.705379963	1.439960003
SDF4	1.703480005	0.709573984
BCKDHB	1.703230023	0.925620019
RPL12	1.702360034	1.096070051
RBM26	1.70212996	1.437019944
SMG1P7	1.701900005	2.426480055

VASP	1.701869965	1.004809976
ACOXL	1.701820016	1.122099996
S100PBP	1.701079965	2.208699942
RAB5C	1.70065999	1.602820039
PIK3C2G	1.700330019	1.729930043
STK10	1.698909998	1.353109956
SCML4	1.698240042	2.148200035
MEF2A	1.69817996	1.52662003
TLN2	1.697980046	0.987554014
MYH13	1.697790027	1.177899957
ARIH1	1.69777	2.038589954
LOC101927060	1.697160006	1.019909978
UNC13C	1.69678998	1.008999944
DNAH14	1.696519971	1.375759959
ABCA4	1.696370006	1.560670018
SMAD6	1.69630003	1.749780059
PDE7B	1.695410013	2.308540106
UTP20	1.695279956	1.559460044
CFAP221	1.695269942	1.852939963
ZNF880	1.69501996	0.819711983
AHCY	1.694759965	2.183330059
NARS1	1.693429947	1.966549993
TTPAL	1.693379998	0.816529989

RPL35	1.692939997	0.775206029
LOC124903248	1.691769958	1.79429996
RAPGEF4	1.691490054	2.245889902
ZDHHC1	1.691329956	2.325900078
DR1	1.691230059	1.24052
SRGAP1	1.691130042	1.547770023
ALDH1L1	1.690420032	1.081689954
ZFP92	1.689520001	2.45498991
TREM1	1.689509988	2.776690006
COMTD1	1.689399958	0.657240987
RPL14	1.689030051	1.093739986
SPNS1	1.688269973	0.831345975
LOC107987069	1.68798995	2.150870085
BACH1	1.687739968	1.274090052
PKP2	1.686910033	0.946861029
TIGD6	1.686800003	1.596869946
CDKN2B	1.686380029	1.385990024
TRIO	1.686310053	0.985728025
DTWD1	1.68603003	1.670789957
LINC02102	1.685860038	1.163120031
SMARCA2	1.685559988	2.133929968
RPS3	1.685179949	0.955936015
MMP9	1.683959961	1.542279959

AKAP7	1.683419943	1.327530026
PTCSC1	1.683109999	0.518621027
ZNF226	1.681280017	0.827672005
TFEB	1.681220055	1.197160006
ZNF43	1.680729985	1.040789962
SSUH2	1.680729985	2.278049946
ASPHD1	1.680130005	1.187839985
SMAD1	1.679479957	1.364349961
YY1AP1	1.679450035	0.899532974
FHOD3	1.678930044	1.992269993
LOC124900478	1.678460002	1.151469946
LOC124901245	1.678159952	1.124089956
PDCD11	1.67809999	1.286929965
RN7SK	1.676990032	1.182229996
LIPE	1.676869988	1.078670025
ZNF462	1.676200032	0.883111
DOK6	1.675860047	2.219490051
GAB1	1.675119996	1.196939945
MAS1	1.674659967	1.43046999
KCNG4	1.67414999	0.757579982
METTL25B	1.673949957	1.889520049
SNX13	1.673920035	1.692569971
ERAP2	1.673650026	1.442989945

LOC124901390	1.673030019	2.052449942
AAMP	1.672719955	0.614224017
CHRNB4	1.672549963	1.460899949
LOC105374056	1.672279954	1.796509981
SCML2	1.670480013	2.628070116
LOC124902215	1.670079947	0.893495977
GAD1	1.669610023	1.214560032
ADGRA3	1.668179989	2.342380047
RALGAPA2	1.668059945	0.997572005
RPIA	1.667870045	1.729629993
SLITRK3	1.667780042	2.075059891
TLE5	1.667410016	0.75721699
TCOF1	1.665869951	1.879490018
NPAP1	1.665689945	1.269680023
LARP4	1.664839983	0.664587975
ZNF528	1.663859963	1.335019946
RPL13	1.66305995	1.291260004
NUCB1	1.662930012	1.720010042
CELF4	1.662770033	1.836899996
ABCC10	1.662449956	1.942770004
EPB41L3	1.661489964	2.137599945
COL15A1	1.660670042	1.692659974
LDLRAD4	1.66031003	0.597938001

SCYL2	1.659999967	1.85837996
CNTN1	1.658759952	1.35947001
LOC105376244	1.658040047	2.470400095
NTAQ1	1.657999992	1.975999951
TP53AIP1	1.65775001	0.932238996
LRRC4C	1.657220006	1.386569977
RPL3	1.657060027	1.153069973
EEF2	1.657050014	1.617370009
LOC105372524	1.656700015	0.978240013
BBX	1.655640006	1.669540048
MKRN1	1.654989958	0.737196028
TCHP	1.654469967	1.089529991
DSCAML1	1.654209971	1.738749981
ARPC3	1.653890014	0.793044984
ATG7	1.653650045	1.201760054
LOC124900946	1.653620005	0.913139999
HPCAL1	1.653450012	1.22676003
PCDH9	1.653429985	0.720669985
AIF1L	1.653249979	0.685639024
ARHGAP1	1.65309	1.64594996
NEFH	1.652539968	1.707450032
DNAH9	1.652359962	1.176939964
UBE2D3	1.652289987	0.954885006

DLK1	1.651870012	0.966695011
POLR3A	1.651419997	2.006880045
CAPN13	1.650210023	1.707069993
ITPKB	1.649760008	1.616340041
LOC107985088	1.649129987	1.451490045
RPL37	1.648759961	0.866093993
LOC124904692	1.648509979	1.349930048
ELAVL4	1.648020029	1.261970043
TSPYL2	1.647899985	1.057100058
AFG1L	1.647340059	1.395140052
HCN1	1.646819949	0.865438998
LOC105376058	1.646479964	1.495730042
E2F1	1.646420002	0.908204019
ZBTB25	1.646090031	1.640709996
FAR1	1.645990014	2.531879902
RFC3	1.645939946	1.200590014
LOC124901239	1.645679951	1.600759983
ABI3	1.645050049	1.249250054
RPS12	1.644899964	0.834136009
LOC124905246	1.644469976	1.492140055
CALM1	1.642850041	1.108049989
FAM117B	1.64278996	2.116980076
SOGA1	1.642660022	2.209409952

ATRX	1.642490029	1.871580005
FBXW10B	1.641459942	1.923310041
UBAP2	1.641450047	1.104969978
RPL29	1.641139984	0.958622992
EPSTI1	1.641129971	1.577980042
CCAR1	1.641049981	1.958189964
STK3	1.640370011	0.94423002
NGFR-AS1	1.640370011	1.386569977
BPIFC	1.640050054	0.691720009
PDE1C	1.639389992	1.262470007
PI4K2A	1.638960004	1.460309982
CAVIN2-AS1	1.63827002	1.00067997
CHST8	1.637310028	1.568289995
PPP1R12A	1.636819959	2.834290028
CAPN8	1.63677001	1.41181004
LOC124900979	1.635820031	2.043459892
INPP4B	1.635290027	1.797950029
CEP41	1.634899974	1.159700036
CARF	1.634140015	2.146219969
XBP1	1.633810043	1.263470054
LOC124907967	1.633479953	1.11097002
GFRA2	1.633329988	1.037019968
COL6A5	1.633280039	1.729650021

HIF1AN	1.633260012	1.645480037
TRS-GCT1-1	1.632940054	0.861905992
TALDO1	1.632750034	1.195359945
SFI1	1.631850004	1.751049995
SLC25A25	1.631829977	1.08059001
LOC105379173	1.631479979	1.526389956
POLR1D	1.631100059	1.861060023
ACOX3	1.631060004	1.383980036
FBXL5	1.630589962	1.848510027
ABTB3	1.630270004	1.715090036
CCDC194	1.62962997	1.212800026
SREK1	1.629320025	1.823109984
LOC124900605	1.629299998	1.527680039
LAMC2	1.628690004	1.800169945
SELENON	1.628190041	1.479220033
LOC107984714	1.628129959	1.624899983
SP3	1.627650023	1.725540042
KRT1	1.627310038	1.326660037
LTF	1.627050042	1.716619968
CTB-99A3.1	1.626850009	1.884260058
CECR3	1.62646997	0.767966986
ZNF33B	1.626219988	1.298689961
KCNB2	1.625929952	1.253659964

PARD3B	1.625399947	0.856477976
SLC4A5	1.625339985	1.448160052
ARPC1B	1.624760032	1.117740035
ARMC8	1.624580026	2.272480011
ANO1	1.624029994	1.694479942
USPL1	1.623510003	0.808247983
ROR1	1.622969985	1.455489993
ANO5	1.622869968	2.144840002
MORF4L1	1.622750044	1.243919969
LOC101929227	1.622460008	1.832430005
SF3A1	1.62203002	1.08818996
MIS18A	1.62184	2.926919937
LOC105373945	1.621549964	2.163680077
ARMC9	1.621530056	1.051200032
RPS15A	1.620910048	0.945650995
TTC7B	1.620820045	1.484899998
RPL38	1.620779991	1.057410002
RPL28	1.620650053	1.063449979
TCP11L2	1.620110035	1.362540007
SCN2A	1.619689941	1.545600057
FRYL	1.618890047	1.091339946
CACNA2D1	1.61875999	1.981969953
ITGB5	1.618180037	2.015919924

PWWP3A	1.617730021	1.615949988
NAV2	1.617499948	1.946460009
DDX5	1.617429972	0.749203026
NALF1	1.617280006	1.325700045
EXO1	1.616700053	1.544190049
PIM1	1.616590023	1.482939959
EZR	1.616009951	0.801048994
LOC158435	1.61590004	1.686139941
EEF1G	1.615730047	1.143540025
ST14	1.615350008	0.894568026
GIMAP7	1.615309954	1.139680028
PTGS1	1.614899993	1.585549951
CHRND	1.614680052	1.104570031
PHIP	1.614140034	2.0666399
PPP1R15A	1.613190055	1.227609992
YBX1	1.613109946	1.104750037
SET	1.611410022	0.837805986
RUFY3	1.611279964	1.730450034
WWC3	1.61105001	1.235759974
UBA5	1.610880017	2.074919939
SNX30	1.610710025	1.570829988
ANKRD17	1.610550046	0.782853007
LOC105370612	1.609259963	2.093300104

ADCK1	1.609060049	1.805259943
RPL18	1.609040022	0.726737976
KMT2A	1.608450055	1.376960039
PAPPA2	1.607759953	0.870495021
MIR99AHG	1.607349992	0.970399976
WNT11	1.607059956	1.522040009
MAN1B1	1.60702002	1.439540029
LOC339685	1.606960058	1.953449965
LOC102724858	1.606830001	1.239539981
RTN4	1.606160045	1.775670052
DNAJC7	1.605530024	1.424379945
PDPR	1.605440021	0.972265005
ABCC9	1.605389953	2.005810022
CR1	1.605020046	2.243609905
DRG1	1.604959965	1.203490019
LOC101927263	1.604670048	1.430539966
NOS1AP	1.603579998	1.771399975
LOC105369559	1.603469968	0.894383013
IRX6	1.603440046	1.527320027
TCTN2	1.603080034	1.502150059
PTGIR	1.602329969	2.096630096
LOC105374140	1.602270007	1.368340015
RALBP1	1.602239966	1.195690036

ANP32E	1.601930022	1.269420028
RGCC	1.601279974	2.044650078
CHMP4B	1.60010004	1.460690022
MAP3K20	1.599730015	1.314239979
BLOC1S3	1.599550009	2.061969995
CLDN2	1.599030018	1.207039952
GBP2	1.598850012	2.985709906
MTR	1.598729968	1.858540058
LOC107986476	1.598690033	2.288680077
POLR1B	1.598629951	1.982980013
IL18BP	1.597669959	1.898830056
IGSF1	1.597599983	2.103470087
ITGAM	1.597219944	2.026329994
LOC107986343	1.596770048	1.316949964
TCF7	1.59660995	1.221179962
ARPC2	1.596410036	0.554854989
UCP2	1.595989943	1.612269998
ABHD14B	1.59592998	0.81449002
ZNF7	1.595849991	1.62311995
SGTA	1.595649958	1.442180037
SAMD9L	1.594820023	2.334549904
USF2	1.594689965	0.947152972
RXRA	1.594570041	1.105029941

DPY30	1.594480038	1.539379954
FAM78B	1.594460011	1.935179949
GOLGA5	1.593960047	0.794852018
INSYN2A	1.592779994	0.630322993
HEMGN	1.592120051	0.681361973
SUGCT	1.590639949	2.090450048
RFX4	1.590569973	1.93422997
FER	1.590250015	2.053380013
SLC8A1	1.590000033	1.139230013
HLCS	1.589969993	2.160759926
KCNQ2	1.589820027	1.618010044
SYNDIG1	1.589589953	2.300120115
C5	1.589529991	2.362560034
RUFY2	1.58912003	2.963119984
CACNA2D2	1.588909984	2.141979933
RPL7	1.587779999	0.889081001
HDGFL2	1.58750999	0.984733999
LOC105370832	1.587229967	1.24416995
LOC107985897	1.586899996	1.685899973
SIDT1	1.586760044	2.076519966
RHOA	1.586689949	0.925763011
TRMT11	1.585790038	0.842522025
UBE2Q2	1.584120035	0.904904008

LOC107984805	1.584030032	1.23100996
TUSC3	1.583760023	0.720577002
DOCK2	1.583150029	1.637740016
LOC124900232	1.583019972	0.791630983
LINC00898	1.582360029	0.74465698
PPP1R12B	1.581830025	0.848233998
TRS-TGA4-1	1.581820011	0.608241022
ILDR1	1.581629992	1.932719946
LDB3	1.581470013	1.60739994
KCND3	1.581329942	1.61717999
LOC124902678	1.581050038	1.376369953
ALDH1L2	1.580970049	1.668280005
FYCO1	1.580880046	1.297610044
WDFY3	1.580749989	1.432659984
ZNF737	1.580629945	1.540660024
GPX4	1.580520034	0.658362985
TRY-GTA6-1_1	1.580450058	1.080010056
GNA13	1.579849958	1.038020015
EPHX1	1.579769969	0.489152014
LOC124904041	1.579640031	2.3692801
QSOX2	1.579110026	1.294540048
SGIP1	1.578580022	2.048340082
LOC124901607	1.577630043	0.86520201

BMPER	1.577329993	1.177379966
SULT1E1	1.576789975	1.451910019
CETP	1.576679945	1.589650035
CARD19	1.575989962	0.788923025
MBD5	1.57585001	1.336959958
SLA	1.575749993	1.758870006
RAB7A	1.575359941	1.030249953
COL4A4	1.574180007	0.976815999
LARGE1	1.57407999	1.151970029
CCNL2	1.574059963	1.214740038
ITPR2	1.573510051	0.80568397
TMEM114	1.573070049	1.339460015
NOP53	1.57257998	1.43532002
SKAP1	1.572340012	1.431270003
LOC105372564	1.572289944	2.595949888
MOB3A	1.571740031	0.944097996
FGD6	1.571630001	1.455029964
UBR3	1.571439981	1.057979941
FAM135B	1.57122004	0.980174005
HDHD5	1.571200013	2.094269991
RYR2	1.570719957	0.93630302
DNMT1	1.569800019	1.376119971
PRKD1	1.569679976	1.580559969

TPT1	1.569380045	1.074290037
HEPH	1.569010019	1.248890042
LOC124904337	1.568510056	1.993379951
SPATA31D1	1.568259954	1.849380016
TCF25	1.568230033	1.010210037
TSKS	1.568220019	0.762320995
UBXN1	1.567260027	0.940961003
LOC102723739	1.566820025	1.371690035
SLC44A5	1.566589952	2.475039959
DST	1.566499949	1.184880018
LOC107985993	1.566030025	2.159009933
LRP2BP	1.564669967	1.505010009
HNRNPK	1.564550042	0.914541006
CCDC77	1.564309955	1.578809977
WDR33	1.564180017	1.250349998
LINC02034	1.563860059	2.537240028
MAGI2-AS3	1.563730001	1.772650003
PARP6	1.563670039	1.586349964
ZNF337	1.562960029	1.623939991
GNPDA1	1.562569976	1.263630033
TRAF3IP2-AS1	1.562520027	2.271509886
HSPA8	1.561360002	0.796476007
ACTR3C	1.561310053	2.220760107

LOC105376176	1.561210036	1.570870042
LOC105375631	1.560840011	1.883229971
SNTB1	1.560400009	0.869490981
CEP95	1.559990048	2.224900007
DENND3	1.559569955	2.074709892
KRT78	1.559470057	1.561179996
BNIP2	1.559430003	2.035550117
CELF3	1.558750033	1.472869992
NACA	1.558519959	0.936088979
ANKFY1	1.557999969	2.005779982
GPR108	1.557989955	1.853850007
FIGNL2	1.557700038	2.473439932
RPLP2	1.556929946	0.891758978
MROH2B	1.556499958	1.586850047
HSD3BP4	1.556439996	1.79321003
CFAP69	1.556020021	1.842910051
ATP11A	1.555889964	1.247439981
HS3ST4	1.55569005	1.918560028
MKNK1	1.555379987	2.587939978
CSRNP3	1.554810047	1.795150042
VLDLR	1.554239988	1.410549998
ZNF213	1.553239942	1.040910006
CLCN1	1.553050041	2.164920092

TTC1	1.552960038	0.510609984
NSD3	1.552729964	1.65083003
COG4	1.552440047	1.075530052
MBNL3	1.552219987	0.942853987
STAB2	1.551839948	1.304720044
PLXNB2	1.55163002	0.815172017
RPL6	1.550830007	0.944954991
SLC5A9	1.550629973	1.243139982
LOC645261	1.550459981	0.537708998
RNF123	1.550449967	0.77442801
BRSK1	1.550430059	2.613029957
EEF1A1	1.550410032	1.208160043
STARD13	1.55024004	1.437700033
LOC105371773	1.550040007	1.407410026
CPOX	1.549900055	1.175089955
PTPRT	1.549479961	2.109339952
SYT9	1.548159957	1.366000056
BUB3	1.547979951	1.824710011
LOC105378867	1.547850013	1.282250047
FAM210B	1.547449946	0.645226002
KAT2B	1.547440052	1.796159983
CFL1	1.547230005	0.947772026
AOPEP	1.546990037	1.044999957

LOC124901058	1.546810031	1.995159984
LOC107986461	1.546550035	2.086630106
PPFIA4	1.546210051	1.367300034
SLC25A35	1.545449972	1.271950006
PTPN21	1.544880033	1.487460017
PDE6B	1.544420004	1.383810043
RPS6	1.544020057	0.709617019
PIP4K2C	1.54392004	2.266609907
SIPA1L1	1.543869972	0.789551973
SAFB2	1.543769956	1.437909961
SLCO5A1	1.543730021	0.960346997
SNRPN	1.542899966	1.909450054
SATB2-AS1	1.542659998	0.705130994
GLI3	1.542029977	1.486719966
IRF2	1.541769981	2.72390008
ADGRF5	1.54152	1.983450055
LOC124902806	1.54144001	1.922420025
WHAMM	1.541200042	2.00970006
BHLHE40	1.539999962	1.076900005
GNAQ	1.539469957	0.754499018
C1orf116	1.538980007	1.514260054
RSRC1	1.536999941	2.539259911
LOC107986015	1.536530018	0.952911973

FAT2	1.535889983	0.930736005
ACO1	1.535480022	1.891450047
LOC105379854	1.535470009	1.377220035
RPL27A	1.535380006	0.816708028
CCNI	1.535320044	0.688767016
EIF2B4	1.535259962	0.589471996
RPL23	1.534770012	0.805167973
MYO5A	1.534530044	2.500469923
TEKT3	1.533460021	1.772359967
ARID1A	1.53234005	1.932299972
CDC20B	1.53192997	1.790590048
ADCY3	1.531780005	1.787430048
GARNL3	1.531479955	0.859376013
RPL11	1.531350017	0.773500979
LOC107985141	1.531190038	0.770277023
SIRPA	1.531159997	1.828089952
LOC107985949	1.530740023	1.663249969
GALNT14	1.530640006	0.766764998
ALDH1A2-AS1	1.530410051	1.763610005
HOXB-AS2	1.530380011	0.868396997
RPS13	1.530240059	1.605949998
LOC124901202	1.529889941	0.578055978
EDF1	1.529719949	0.884091973

CARMAL	1.529099941	2.146140099
TTC21B	1.528270006	1.615829945
PKHD1	1.528130054	1.035169959
PROSER3	1.527909994	1.957589984
MTPN	1.527809978	1.006010056
ADGRF2	1.527799964	2.385580063
RPL23A	1.526859999	1.025470018
TFDP1	1.526479959	1.348199964
STS	1.526090026	0.83188802
LOC105371271	1.525889993	0.966584027
MSN	1.524680018	1.317399979
NDST3	1.524559975	1.409549952
LOC124901725	1.524350047	0.878169
LINC02822	1.524260044	0.764826
LINC01566	1.520640016	0.762579978
CC2D1B	1.520059943	0.655734003
RAB11FIP2	1.519880056	1.812070012
CNTN2	1.519170046	2.611530066
LOC105372210	1.519090056	2.379390001
RPSA	1.517670035	1.335909963
CBX3	1.517019987	1.469390035
PABPC1	1.516780019	0.949334025
ITGAE	1.516630054	1.620609999

LOC124902462	1.516459942	1.584609985
DPAGT1	1.516350031	2.639590025
INSR	1.515789986	0.779576004
ANKRD23	1.515730023	1.25387001
LOC124903051	1.514999986	1.156489968
LOC124903832	1.514879942	1.801949978
NRCAM	1.51485002	1.22499001
TTI1	1.514709949	2.158370018
RPLP1	1.514350057	1.299999952
LTBP3	1.514299989	0.727120996
LOC101927345	1.514209986	1.897959948
NUDCD3	1.51402998	2.552459955
RPTOR	1.513890028	1.859760046
TGM7	1.513679981	1.61274004
RPL5	1.513579965	0.892542005
NLGN1	1.512789965	1.243899941
BLTP2	1.512699962	1.8671
CCL22	1.512580037	1.424000025
MECP2	1.512549996	1.311329961
GTDC1	1.512300014	1.294620037
NEU3	1.512199998	2.292370081
EML5	1.511100054	1.445739985
LOC105370567	1.510969996	1.301900029

SLC4A1	1.510939956	1.708430052
LOC102724036	1.510879993	1.408030033
RAB13	1.510489941	0.918568015
DROSHA	1.510460019	2.651809931
MAPRE2	1.510259986	0.970966995
EGFEM1P	1.509950042	1.359779954
ELF1	1.509940028	1.469220042
JMY	1.509469986	2.459280014
LOC124903379	1.509420037	0.673134983
ACSBG1	1.509320021	0.854192972
RIC8B	1.509269953	1.38883996
PRG4	1.509140015	1.05934
LOC105369896	1.50880003	1.102519989
WDTC1	1.508489966	1.780500054
FLJ42393	1.508080006	1.549769998
LOC124904195	1.507969975	1.682420015
OTX1	1.507799983	2.090389967
LOC124901403	1.505990028	2.096839905
RAD51AP2	1.505749941	1.25177002
PTPN13	1.504819989	1.606340051
RAP2A	1.504639983	1.031890035
NES	1.504510045	0.899766982
LOC124904287	1.504500031	1.190709949

ZNF789	1.503990054	1.614949942
MOK	1.503919959	1.528560042
HECTD3	1.503640056	1.234500051
MYT1L	1.503440022	1.219889998
UPF1	1.502159953	1.227970004
EIF4G2	1.501760006	1.53034997
RPL8	1.501749992	1.027750015
VAPA	1.501019955	0.825851977
C1QTNF7	1.500839949	1.029899955
RPL32	1.500249982	0.622664988
STIP1	1.500130057	0.952486992
PRKACB	1.499979973	1.463019967
C8A	1.499600053	1.777220011

Supplementary Table 6. miRNA PLSR VIP Scores

Primary ID	VIP Score	VIP ± Standard Error
hsa-miR-483-5p	2.192500114	1.917129993
hsa-miR-106b-5p	2.144730091	1.726940036
hsa-miR-223-5p	2.09533	2.129800081
hsa-miR-142-5p	2.021049976	1.192289948
hsa-let-7b-5p	1.932770014	1.773460031
hsa-miR-199b-5p	1.883020043	1.184520006
hsa-miR-195-5p	1.788630009	2.913019896
hsa-let-7g-5p	1.769490004	2.0697999
hsa-miR-340-5p	1.724730015	1.29005003
hsa-miR-30e-5p	1.643290043	1.45994997
hsa-miR-17-5p	1.625470042	1.74477005
hsa-miR-191-5p	1.623669982	2.587270021
hsa-miR-4433b-5p	1.621269941	2.066499949
hsa-miR-744-5p	1.605020046	1.960180044
hsa-miR-10b-5p	1.581640005	1.79460001
hsa-miR-1306-5p	1.580469966	0.531952977
hsa-miR-3613-5p	1.562330008	0.935923994
hsa-miR-382-5p	1.497280002	1.767950058
hsa-miR-451a	1.427970052	2.658449888
hsa-miR-10a-5p	1.368579984	1.972360015
hsa-miR-30d-5p	1.330899954	0.957104981

hsa-miR-1277-5p	1.31238997	1.122650027
hsa-miR-224-5p	1.303959966	1.984099984
hsa-miR-424-5p	1.285740018	1.172780037
hsa-miR-146a-5p	1.284500003	2.916090012
hsa-miR-18a-5p	1.280189991	0.757277012
hsa-miR-93-5p	1.265949965	2.659879923
hsa-miR-192-5p	1.256389976	1.804399967
hsa-miR-584-5p	1.152590036	1.647369981
hsa-miR-769-5p	1.133659959	1.343389988
hsa-miR-196b-5p	1.115659952	2.250309944
hsa-let-7c-5p	1.104650021	1.50375998
hsa-miR-548d-5p	1.046560049	2.088010073
hsa-miR-30a-5p	1.044870019	1.846789956
hsa-miR-431-5p	1.041280031	2.233370066
hsa-miR-337-5p	1.027230024	1.559939981
hsa-miR-204-5p	0.999062002	1.193079948
hsa-miR-182-5p	0.98842603	2.495130062
hsa-miR-345-5p	0.918908	2.076930046
hsa-miR-4732-5p	0.906892002	1.877570033
hsa-miR-324-5p	0.856579006	2.146680117
hsa-miR-151a-5p	0.848545015	1.050879955
hsa-let-7a-5p	0.822048008	1.570680022
hsa-miR-125a-5p	0.802074015	2.093230009

hsa-miR-339-5p	0.796747029	2.001709938
hsa-miR-98-5p	0.795880973	1.175750017
hsa-let-7e-5p	0.792940021	2.041090012
hsa-miR-335-5p	0.781997979	1.260849953
hsa-let-7d-5p	0.767194986	1.725000024
hsa-miR-125b-5p	0.750648975	2.247489929
hsa-miR-199a-5p	0.749957025	2.040719986
hsa-miR-432-5p	0.729806006	1.324550033
hsa-miR-532-5p	0.720031977	2.816230059
hsa-miR-96-5p	0.709146023	1.499140024
hsa-miR-15a-5p	0.708950996	2.458990097
hsa-miR-20a-5p	0.692247987	2.389359951
hsa-miR-374b-5p	0.688943982	1.635599971
hsa-miR-190a-5p	0.686191022	1.683150053
hsa-miR-484	0.676500976	1.738129973
hsa-miR-139-5p	0.660528004	2.037220001
hsa-miR-21-5p	0.65247798	2.391520023
hsa-miR-30b-5p	0.650629997	1.94441998
hsa-miR-411-5p	0.625972986	1.61669004
hsa-miR-155-5p	0.625055015	1.220070004
hsa-miR-186-5p	0.62412101	2.388279915
hsa-miR-486-5p	0.611409009	2.39880991
hsa-miR-185-5p	0.589654028	2.414000034

hsa-let-7i-5p	0.573723972	1.43295002
hsa-miR-16-5p	0.565711021	1.232689977
hsa-miR-942-5p	0.564671993	1.612570047
hsa-miR-181b-5p	0.540405989	2.231620073
hsa-miR-425-5p	0.537827015	1.234259963
hsa-miR-379-5p	0.534015	1.528599977
hsa-miR-576-5p	0.477553993	2.751909971
hsa-miR-134-5p	0.476496011	1.02670002
hsa-miR-503-5p	0.474040985	1.61421001
hsa-miR-574-5p	0.470656008	1.940179944
hsa-miR-7-5p	0.467020988	1.968009949
hsa-miR-660-5p	0.444916993	1.828549981
hsa-miR-664a-5p	0.425127	0.955247998
hsa-miR-194-5p	0.421038985	1.249199986
hsa-miR-26b-5p	0.402348995	1.927379966
hsa-miR-150-5p	0.376558989	1.958279967
hsa-miR-140-5p	0.367195994	1.429010034
hsa-miR-629-5p	0.343338996	1.874920011
hsa-miR-146b-5p	0.339964002	1.229859948
hsa-miR-99b-5p	0.325006008	1.528949976
hsa-miR-148b-5p	0.324443996	1.189010024
hsa-miR-183-5p	0.318174005	0.875671983
hsa-miR-11400	0.29718399	1.273939967

hsa-miR-9-5p	0.27629301	1.653499961
hsa-miR-122-5p	0.270062	1.815760016
hsa-let-7f-5p	0.265585989	1.411999941
hsa-miR-32-5p	0.265500993	1.374600053
hsa-miR-144-5p	0.264605999	1.438760042
hsa-miR-20b-5p	0.252548009	1.156309962
hsa-miR-423-5p	0.228980005	1.322080016
hsa-miR-181a-5p	0.199343994	1.068989992
hsa-miR-26a-5p	0.179317996	1.033329964
hsa-miR-145-5p	0.173539996	1.194260001
hsa-miR-30c-5p	0.172466993	0.486447006
hsa-miR-126-5p	0.156872004	1.647719979
hsa-miR-15b-5p	0.154312	1.953420043
hsa-miR-625-5p	0.140019998	0.695083976
hsa-miR-369-5p	0.104667	1.69509995
hsa-miR-361-5p	0.067826897	1.309159994
hsa-miR-374a-5p	0.0424488	1.072039962

Supplementary Table 7. RT-qPCR Primers

Target	Forward (F)	Reverse (R)
ENAH	5' - TCTATCACCATACAGGCAACAAC - 3'	5' - GCACAGTTTATCACGACCTGA - 3'
EXOC4	5' - AAAGGGTCGCCTTGAAGAAGC - 3'	5' - TCGTCAATTCTGTGTAGTGCTG - 3'
GAPDH	5' - GTCTCCTCTGACTTCAACAGCG - 3'	5' - ACCACCCTGTTGCTGTAGCCAA - 3'
GEMIN5	5' - CCTCCGTCTTCCTTGTCCG - 3'	5' - CAGAGACCCTTTCGGTGTGTC - 3'
GFRA1	5' - CCAAAGGGAACAACTGCCTG - 3'	5' - CGGTTGCAGACATCGTTGGA - 3'
GLE1	5' - TTGAAGGCCCTACGCAGTTC - 3'	5' - ACCACCCATCCAGAATAAGAAGA - 3'
MAPK6	5' - TGTGTGGAATCAAGCACCTTC - 3'	5' - AGGCGTCATCATAAAACTCGTTC - 3'
NFATC2	5' - GAGCCGAATGCACATAAGGTC - 3'	5' - CCAGAGAGACTAGCAAGGGG - 3'
NFKB1	5' - AACAGAGAGGATTTCGTTTCCG - 3'	5' - TTTGACCTGAGGGTAAGACTTCT - 3'
PPP2R5B	5' - CTCCTCTCAGTTCCGCTATCA - 3'	5' - GCCACACAGTCCAAGAAGTCAA - 3'
SHC3	5' - CTTGGGGTGCATTGAAGTTCT - 3'	5' - GGCTTCCCTGGTAATTTGTGTT - 3'
